# A Polyphasic and Taxogenomic Evaluation Uncovers *Arcobacter cryaerophilus* as a Species Complex That Embraces Four Genomovars

**DOI:** 10.3389/fmicb.2018.00805

**Published:** 2018-04-27

**Authors:** Alba Pérez-Cataluña, Luis Collado, Oscar Salgado, Violeta Lefiñanco, María J. Figueras

**Affiliations:** ^1^Unit of Microbiology, Department of Basic Health Sciences, Faculty of Medicine and Health Sciences, IISPV, University Rovira i Virgili, Reus, Spain; ^2^Faculty of Sciences, Institute of Biochemistry and Microbiology, Universidad Austral de Chile, Valdivia, Chile; ^3^Laboratory of Microbial Ecology of Extreme Systems, Department of Molecular Genetics and Microbiology, Pontificia Universidad Católica de Chile, Santiago, Chile

**Keywords:** *Arcobacter cryaerophilus*, *is*DDH, ANI, MLPA, genomovar

## Abstract

The species *Arcobacter cryaerophilus* is found in many food products of animal origin and is the dominating species in wastewater. In addition, it is associated with cases of farm animal and human infectious diseases,. The species embraces two subgroups i.e., 1A (LMG 24291^T^ = LMG 9904^T^) and 1B (LMG 10829) that can be differentiated by their 16S rRNA-RFLP pattern. However, some authors, on the basis of the shared intermediate levels of DNA-DNA hybridization, have suggested abandoning the subgroup classification. This contradiction indicates that the taxonomy of this species is not yet resolved. The objective of the present study was to perform a taxonomic evaluation of the diversity of *A. cryaerophilus*. Genomic information was used along with a Multilocus Phylogenetic Analysis (MLPA) and phenotypic characterization on a group of 52 temporally and geographically dispersed strains, coming from different types of samples and hosts from nine countries. The MLPA analysis showed that those strains formed four clusters (I–IV). Values of Average Nucleotide Identity (ANI) and *in silico* DNA-DNA Hybridization (*is*DDH) obtained between 13 genomes representing strains of the four clusters were below the proposed cut-offs of 96 and 70%, respectively, confirming that each of the clusters represented a different genomic species. However, none of the evaluated phenotypic tests enabled their unequivocal differentiation into species. Therefore, the genomic delimited clusters should be considered genomovars of the species *A. cryaerophilus*. These genomovars could have different clinical importance, since only the cluster I included strains isolated from human specimens. The discovery of at least one stable distinctive phenotypic character would be needed to define each cluster or genomovar as a different species. Until then, we propose naming them “*A. cryaerophilus gv. pseudocryaerophilus*” (Cluster I = LMG 10229^T^), “*A. cryaerophilus gv. crypticus*” (Cluster II = LMG 9065^T^), “*A. cryaerophilus gv. cryaerophilus”* (Cluster III = LMG 24291^T^) and “*A. cryaerophilus gv. occultus*” (Cluster IV = LMG 29976^T^).

## Introduction

The genus *Arcobacter*, within the family *Campylobacteraceae*, was proposed by Vandamme et al. (1991) to reclassify two species that were, at that time, assigned to the genus *Campylobacter* i.e., *Campylobacter nitrofigilis* (*Arcobacter nitrofigilis*, that was selected as the representative or the type species of the genus) and *Campylobacter cryaerophila* (now *Arcobacter cryaerophilus*). The phenotypic characteristics that differentiate *Campylobacter* and *Arcobacter* are the ability of the latter to grow in aerobic conditions and at lower temperatures (Vandamme et al., 1991; Collado and Figueras, 2011).

Using more than 4000 genomes, Waite et al. (2017) recently analyzed the 16S and 23S rRNA genes and 120 protein sequences and as a result they moved the *Epsilonproteobacteria* to the phylum level with the name Epsilonbacteraeota. In addition, they created a new family *Arcobacteraceae* that includes only the genus *Arcobacter*. Currently, the genus *Arcobacter* includes 27 species (Park et al., 2016; Whiteduck-Léveillée et al., 2016; Diéguez et al., 2017; Figueras et al., 2017; Tanaka et al., 2017; Pérez-Cataluña et al., 2018), four of which have been linked with human disease: *Arcobacter butzleri, A. cryaerophilus, A. thereius*, and *A. skirrowii* (Collado and Figueras, 2011; Figueras et al., 2014; Ferreira et al., 2015). The species *A. cryaerophilus* has been found in many food products of animal origin (like poultry, pork, lamb, and seafood and in dairy food processing facilities (Collado et al., 2008; Collado and Figueras, 2011).

On the basis of the different Restriction Fragment Length Polymorphism (RFLP) of the 16S and 23S rRNA genes, Kiehlbauch et al. (1991) and Vandamme et al. (1992) divided the species *A. cryaerophilus* into two subgroups, subgroup 1 or 1A and subgroup 2 or 1B (from here on we will call them subgroups 1A and 1B), represented by strains LMG 24291^T^ (= LMG 9904^T^) and LMG 10829, respectively. Additionally, it was demonstrated that the two subgroups showed different whole-cell protein and fatty acid contents (Vandamme et al., 1992) and clustered apart by their Amplified Fragment Length Polymorphism (AFLP) patterns (On et al., 2003). A 16S rDNA-RFLP identification method established the separation of the subgroups on the basis of their restriction patterns (Figueras et al., 2008). Despite strains belonging to both subgroups having been found at the same time in animal and human clinical samples and in food products, 1B is generally much more frequently found than 1A (Collado and Figueras, 2011 and references therein). In 2010, Debruyne et al. (2010) reassessed the taxonomy of these two subgroups of *A. cryaerophilus* using 59 strains isolated mainly from aborted animals (74% of the strains) and human faces (19%). The clustering of the strains obtained by AFLP and by the phylogenetic analysis of the *cpn60* gene, together with the shared intermediate levels of DNA-DNA hybridization observed between the strains lead the authors to conclude that despite *A. cryaerophilus* having a complex taxonomy, the subgroup nomenclature should be abandoned (Debruyne et al., 2010). Furthermore, it was considered that the type strain (LMG 24291^T^ = LMG 9904^T^) of *A. cryaerophilus* was not representative of the species because it corresponded with the less abundant 1A subgroup. They therefore proposed that it should be changed for the strain LMG 10829, representative of subgroup 1B (Debruyne et al., 2010). However, a recent metagenomic analysis of *Arcobacter* populations recovered from sewage samples of the wastewater treatment plant in the city of Reus (Spain) and from various cities of the United States gave evidence that both *A. cryaerophilus* subgroups (1A and 1B) were dominating in this environment (Fisher et al., 2014). In addition, a different prevalence of the two *A. cryaerophilus* subgroups was found depending on the wastewater temperature, 1B dominating in wastewater samples with temperatures above 20°C. Fisher et al. (2014) concluded that this finding is relevant because understanding the ecological factors that affect the fate of *Arcobacter* spp. in wastewater may help to better understand the risks associated with these emerging pathogens. The latter study showed that both subgroups of *A. cryaerophilus* were abundant and represented two different ecotypes. Therefore, based on those findings, a new polyphasic re-evaluation of the taxonomic diversity of this species is required. The aim of the present study was to investigate the taxonomy of *A. cryaerophilus*, evaluating strains from 9 different countries recovered from wastewater, different types of shellfish, human faces and various types of animal samples (feces, various viscera from fetuses, uterus, and milk). To our knowledge, this is the most diverse collection of strains of this species studied so far. The polyphasic study involved a phylogenetic analysis of the sequences of the 16S and 23S rRNA genes and of several housekeeping genes, an analysis of 13 genomes (7 of which were obtained in this study) from a representative strains and a phenotypic characterization.

## Materials and methods

### Strains used in this study

The study included a total of 52 strains that were widely distributed, both geographically and by the type of sample from which they were isolated that, included different host species (humans, pigs, cow, deer, clams, etc.) and environments (water, milk, reclaimed water etc.) as show in Table 1. Six strains possessed their genomes available at the GenBank database, 36 were field isolates from different sources and countries collected over a broad time frame (1985–2013) and 10 strains were from the BCCM/LMG Bacteria Culture Collection (Table 1). Among the latter was the type strain of *A. cryaerophilus* LMG 24291^T^ that corresponds to subgroup 1A and the reference strain LMG 10829 of the subgroup 1B (Table 1). The 46 strains were re-evaluated or ascribed to subgroups 1A or 1B using the 16S rDNA-RFLP method described by Figueras et al. (2008, 2012). The method consists of the digestion of an amplified fragment (1026 bp) of the 16S rRNA gene with the enzyme *Mse*I, which produces a pattern with different band sizes for subgroup 1A (395, 216, 143, 138 bp) and for subgroup 1B (365, 216, 143, and 138 bp). The RFLP patterns of the six genomes from the GenBank database (genomes L397 to L401 and L406) were obtained by an *in silico* simulation of the enzymatic digestion using GeneQuest software (DNASTAR, USA). When a different pattern from that expected for *A. cryaerophilus* was obtained, it was compared with those patterns described for the type strains of all the *Arcobacter* species by Figueras et al. (2008, 2012). In addition the identity of the strains were confirmed by sequencing the *rpoB* gene using primers and conditions described in other studies (Collado et al., 2009; Levican et al., 2015).

**Table 1:** Strains used (*n* = 52) in this study included field isolates, the type and reference collection strains of the species *A. cryaerophilus* and genomes from the NCBI database^a^ and 7 obtained in this study^b^ (accession numbers in Table 2).

**Country**	**Strain**	**Source**	**Isolation year**	**16S-RFLP Pattern**	**Cluster**
Brazil	F196	Aborted porcine fetus	1997	1B	I
Brazil	UF1T	Uterus, sow	1997	1B	I
Brazil	UF2T	Uterus, sow	1997	1B	I
Brazil	UPER3	Uterus, sow	1997	1B	I
Canada	LMG 10229^b^	Kidney, aborted porcine fetus	1990	1B	I
Canada	LMG 10241	Kidney, aborted porcine fetus	1990	1B	I
Canada	LMG 10210^b^	Aborted bovine fetus	1990	1B	IV
Canada	L397^a^	Wastewater	2008	1B	I
Canada	L398^a^	Water	2008	1B	I
Canada	L399^a^	Wastewater	2008	1B	I
Canada	L400^a^	Wastewater	2008	1B	I
Canada	L401^a^	Goose feces	2009	1B	I
Canada	L406^a^	Water	2008	1B	I
Chile	AB3A	Abomasum, aborted bovine fetus	2011	1B	I
Chile	AB74A	Abomasum, aborted bovine fetus	2013	1B	I
Chile	AO2A	Lungs, aborted ovine fetus	2011	1B	I
Chile	AL 20-1	Clam	2011	1B	II
Chile	CV-152	Feces, deer	2013	1A	III
Chile	CV-2101	Feces, deer	2013	1A	III
Chile	EMU-3	Feces, emu	2013	1A	III
Chile	FE7	Feces, chicken	2005	Abutz	II
Chile	HHS 118A	Feces, asymtomatic human	2013	1B	I
Chile	HHS 133A	Feces, asymtomatic human	2013	1B	I
Chile	HHS 188A	Feces, asymtomatic human	2013	1B	I
Chile	HHS 191A	Feces, asymtomatic human	2013	1B	I
Chile	HHS 205A	Feces, asymtomatic human	2013	1B	I
Chile	MC 2-2	Surf clam	2011	NP	I
Chile	MCV 42-1	Feces, cow	2011	1B	I
Chile	ME 15-4	Mussel	2011	Abutz	II
Chile	NAV 15-1	Razor clam	2011	1A	IV
Chile	NAV12-2	Razor clam	2011	NP	I
Chile	NB14A	Jejunum, calf	2011	1B	I
Costa Rica	14 PHA	Viscera, chicken	2011	1B	I
Costa Rica	20 PHF	Viscera, chicken	2011	1B	I
Ireland	LMG 24291^Tb^	Brain, aborted bovine fetus	1985	1A	III
Ireland	LMG 9065^b^	Placenta, aborted ovine fetus	1989	1A	II
Ireland	LMG 9861^b^	Peritoneum, aborted bovine fetus	1990	1B	I
Ireland	LMG 9863^b^	Placenta, aborted ovine fetus	1990	Abutz	II
Ireland	LMG 29976^b^	Eye, aborted porcine fetus	1990	1A	IV
Ireland	LMG 9871^b^	Kidney, aborted bovine fetus	1990	Abutz	II
Italy	284/1	Cow milk	2012	1B	I
Italy	BUF3	Buffalo milk	2012	1B	I
Italy	FEBU4	Feces, buffalo	2012	1B	I
New Zealand	8749401	Diarrhoeic feces, human	2008	1B	I
New Zealand	8756347	Diarrhoeic feces, human	2008	1B	I
Spain	8122333	Diarrhoeic feces, human	2012	1B	I
Spain	RW15-1	Reclaimed water	2013	1A	IV
Spain	RW17-4	Reclaimed water	2013	1A	IV
Spain	RW25-5	Reclaimed water	2013	1A	I
Spain	RW33-8	Reclaimed water	2013	1A	I
Spain	RW45-3	Reclaimed water	2013	1A	IV
USA	LMG 10829	Human blood	1990	1B	I

### Phylogenetic analysis

A Multilocus Phylogenetic Analysis (MLPA) was carried out by amplifying and sequencing 4 housekeeping genes (*gyrB, rpoB, atpA*, and *cpn60*) following protocols described by Levican Asenjo (2013). In addition, these genes and the 16S and 23S rRNA genes were extracted from the 7 obtained genomes and from the 6 downloaded from the GenBank database. Accession number or locus tag of each gene and strain are show in Supplementary Table S1. Genes were aligned (Supplementary Figure S4) using CLUSTALW (Larkin et al., 2007) implemented in MEGA 6 software (Tamura et al., 2013). The same software was used for the phylogenetic analysis using Neighbor-Joining (NJ) algorithm (Kimura, 1980; Saitou and Nei, 1987) and the bootstrap support for individual nodes was calculated with 1,000 replicates.

### Whole genome sequencing and analysis

The genome sequence of the type strain of *A. cryaerophilus* (LMG 24291^T^) and of six additional strains (LMG 10229^T^, LMG 9861, LMG 9065^T^, LMG 9871, LMG 29976^T^, and LMG 10210) representative of the different MLPA clusters were obtained in the present study using Illumina MiSeq platform (San Diego, CA, USA). The genomic DNA was extracted from pure cultures using the Easy-DNA™ gDNA Purification kit (Invitrogen, Madrid, Spain). Genomic libraries were prepared with the Nextera® XT DNA Sample Preparation Kit (Illumina) following manufacturer's instructions. Genome assembly was carried out with the SPAdes 3.9 (Nurk et al., 2013) and the CGE assemblers (Larsen et al., 2012) and the best results were selected for further analysis. Assembled genomes were annotated using Prokka v1.11 software (Seemann, 2014). Additionally, the protein-encoding sequences (CDS) were annotated using the Rapid Annotation Subsystem Technology (RAST) (Aziz et al., 2008) and the PATRIC server v3.5.2. (Wattam et al., 2017). The general characteristics derived from the NCBI Prokaryotic Genome Automatic Annotation Pipeline (PGAAP) and described for the 13 genomes (6 from the GenBank database and 7 from this study) were: genome size (Mb), number of contigs, N50 (bp), G+C content (%) and the number of predicted CDS. Furthermore, the genomes were compared by the Average Nucleotide Identity (ANI) and the *in silico* DNA-DNA hybridization (*is*DDH) indices using OrthoANI (Lee et al., 2015) and Genome-to-Genome Distance Calculator software (Meier-Kolthoff et al., 2013), respectively.

Additionally, a phylogenetic analysis of the 13 genomes (LMG 24291^T^, LMG 10229^T^, LMG 9861, L397-L401, L406, LMG 9065^T^, LMG 9871, LMG 29976^T^, and LMG 10210) was carried out using the Maximum Likelihood estimation using RAxML (Stamatakis, 2014) with the pipeline implemented in the PATRIC server (Wattam et al., 2017). The genome of *A. trophiarum* LMG 25534^T^was used as outgroup. As a first step, the phylogeny was constructed using a set of homologous proteins identified with BLASTp (Boratyn et al., 2013) and clustered with the Markov Cluster Algorithm (MCL) (Dongen, 2000). The second step was an alignment of the protein set using MUSCLE (Edgar, 2004) and the Hidden Markov Models (HMM) were constructed with HMMER tools (Eddy, 1998).

### Virulence and antibiotic resistance genes

Virulence genes were searched by BLASTn analysis with default parameters using the Virulence Factors of Pathogenic Bacteria Database (VFDB) (Chen et al., 2005), Victors Database (University of Michigan, USA) and PATRIC_VF (Wattam et al., 2017). Antibiotic resistance genes were searched using the Antibiotic Resistance Database (ARDB) (Liu and Pop, 2009) and the Comprehensive Antibiotic Resistance Database (CARD) (Jia et al., 2017). The five mentioned databases are included at the Specialty Genes tool available at the PATRIC server (Wattam et al., 2017). Furthermore, the Antibiotic Resistance Gene-Annotation database (ARG-ANNOT) (Gupta et al., 2014) was also used to search antibiotic resistance genes by BLASTp analysis using default parameters and the database ARG-ANNOT AA V3 (March 2017). Virulence and resistance mechanisms were also searched for with RAST (Aziz et al., 2008) and PATRIC servers (Wattam et al., 2017). Additionally, genes related with the virulence of *Arcobacter* (Collado and Figueras, 2011; Douidah et al., 2012; Levican et al., 2013a) were searched for with BLASTn using sequences obtained from GenBank and from the annotated *Arcobacter* genomes of *A. butzleri* RM4018, *A. nitrofigilis* DSM 7299 and *Arcobacter* sp. L. The genes studied were *cadF* and *cj1349*, which encode two fibronectin binding proteins; *ciaB* encodes the invasion protein CiaB, *mviN* gene related to peptidoglycan synthesis; *pldA* gene encodes a phospholipase; *tlyA* gene codifies for a hemolysine; *hecB* related to hemolysis activation; *hecA* gene that encodes an adhesion protein and finally the gene *irgA* that codifies an iron-regulated outer membrane protein (Collado and Figueras, 2011; Douidah et al., 2012; Levican et al., 2013a). The accession number or locus tag of those genes are show in Supplementary Table S2. A phylogenetic analysis was conducted using the three virulence genes (*cj1349, mviN*, and *pldA*) present in all the studied genomes to evaluate their genetic relatedness and evolution.

### Comparison of the genome derived metabolic and phenotypic information

The genomes of the seven representative strains from each cluster (LMG 10229^T^, LMG 9861, LMG 9065^T^, LMG 9871, LMG 24291^T^, LMG 29976^T^, and LMG 10210) were compared using the Functional Comparison Tool implemented in the Seed Viewer (Overbeek et al., 2014). This software uses the protein sequences of each compared genome annotated with RAST (Aziz et al., 2008) and reconstructs the metabolic pathways. On the other hand, the phenotypic traits derived from each genome were obtained with Traitar software (Weimann et al., 2016) using the protein annotations obtained with Prokka v1.2 (Seemann, 2014). This software infers phenotypic traits using data from the Global Infectious Disease and Epidemiology Online Network (GIDEON) and from the Bergey's Systematic Bacteriology (Goodfellow et al., 2012). The software works with a total of 67 traits that embrace different microbiological or biochemical characteristics involved in enzyme activity, growth, oxygen requirements, morphology, and hydrogen sulfide production (Weimann et al., 2016).

### Phenotypic characterization

Phenotypic characterization of the 46 strains included 9 tests recommended in the guidelines for defining new species of the family *Campylobacteraceae* (Ursing et al., 1994; On et al., 2017) and 7 additional tests used in the description of other *Arcobacter* spp. (Donachie et al., 2005; Houf et al., 2005). Most of these tests were chosen using as a criterion the biochemical tests that gave variable results for both *A. cryaerophilus* subgroups in the previous study by On (1996), in which a total of 67 phenotypic tests were analyzed from 9 and 10 strains of subgroups 1A and 1B, respectively. Growth conditions on blood agar were tested (BD Difco, NJ, USA) at 37° and 42°C at three different atmospheres: aerobic, microaerobic, and anaerobic conditions. The biochemical properties were tested at 30°C in aerobic conditions for the 46 strains using positive and negative controls in parallel for each specific test. To evaluate inter laboratory reproducibility, the strains LMG 9065, LMG 9861, LMG 9871, LMG 10229 and LMG 24291^T^were tested in parallel in two different laboratories in different countries (Chile and Spain).

## Results and discussion

### Molecular identification and phylogeny

Table 1 shows that 46 of the 52 strains gave RFLP patterns defined by Figueras et al. (2008) for *A. cryaerophilus* and 4 showed the one for *A. butzleri* (FE7, ME15-4, LMG 9863, and LMG 9871). However, strains NAV12-2 and MC2-2 produced a new RFLP pattern different to the described ones (Figueras et al., 2008, 2012). From the 46 strains that gave the pattern of *A. cryaerophilus*, 34 gave the pattern of the subgroup 1B (including the in *silico* simulated patterns obtained from the 16S rRNA genes of the 6 GenBank genomes L397- L401 and L406) and 12 the one of the subgroup 1A. This demonstrated once more that subgroup 1B is more abundant than 1A, in agreement with results of previous studies (Debruyne et al., 2010; Collado and Figueras, 2011; Fisher et al., 2014). As Figueras et al. (2012) explained when describing the 16S rDNA-RFLP identification method, different RFLP patterns from those expected for the *Arcobacter* spp. can obtained for new species or might be due to the existence of a mutation on the targeted site of the endonucleases in a known species. The former occurred for instance in *A. mytili* (Collado et al., 2009) and *A. molluscorum* (Figueras et al., 2011a) among other species (Figueras et al., 2011b; Levican et al., 2012, 2013b, 2015). Mutations at the binding site of the endonuclease *MseI* were described in the strains LMG 9863 and LMG 9871 (used in this study, Table 1), but in this case instead of resulting in a new pattern they were responsible for generating the pattern for *A. butzleri* instead of *A. cryaerophilus* (Figueras et al., 2012).

**Figure 1:**
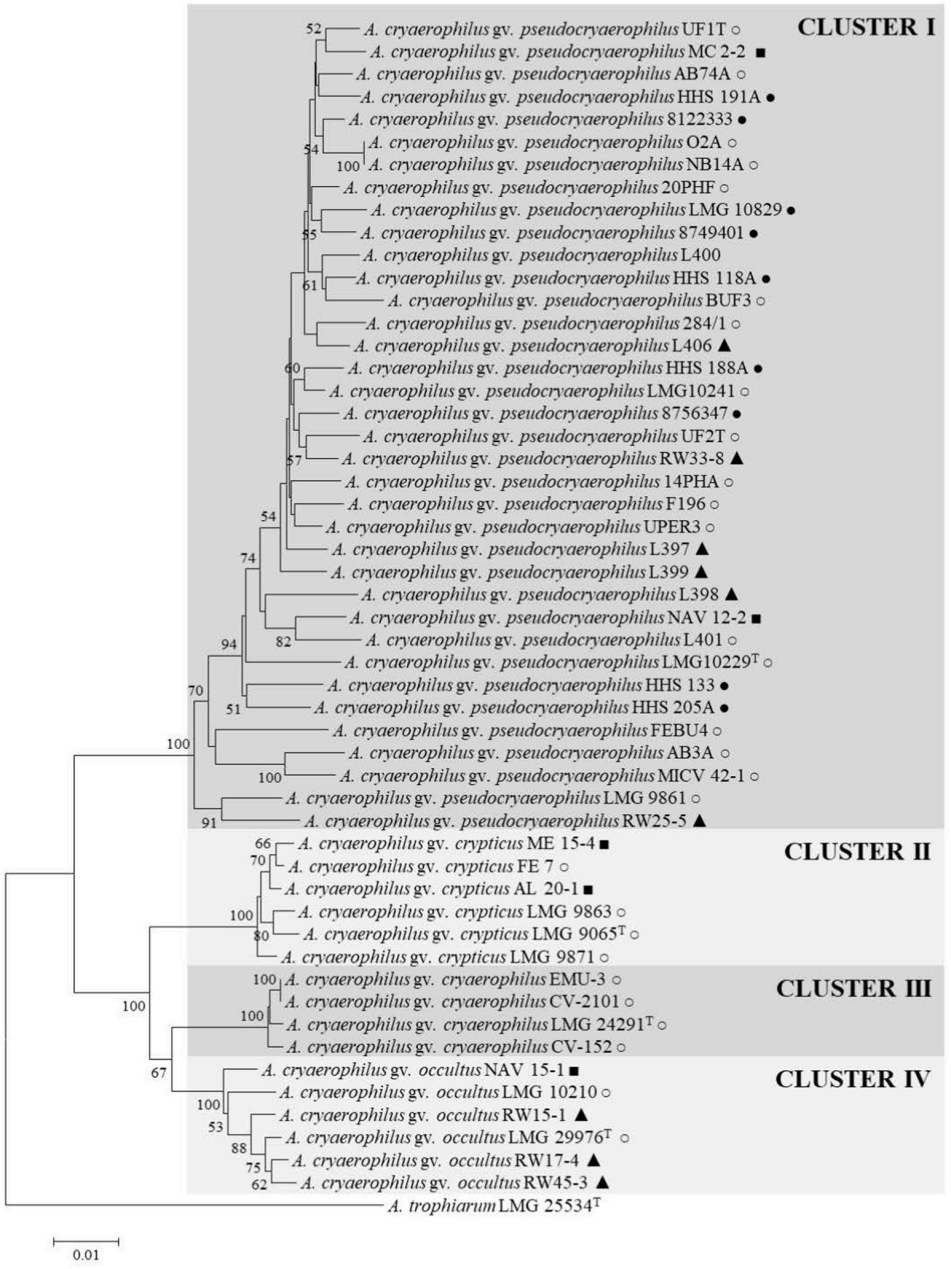
Neighbor joining tree based on the concatenated sequences of *gyrB, rpoB, atpA*, and *hsp60* (2,408 bp) genes showing the distribution of the 52 strains in four clusters. Bootstrap values (>50%) based on 1,000 replications are shown at the nodes of the tree. Bar indicates 1 substitutions per 100 bp. Isolation source: •, Human; ◦, Animal; ■, Shellfish; ▴, Water.

The MLPA with the concatenated sequences (2,408 bp) of the four housekeeping genes (*gyrB, rpoB, atpA*, and *cpn60*) of the 52 strains showed that they grouped into four main clusters (Figure 1). Cluster I had 36 strains, most of them (88.8%) from the subgroup 1B, and included the reference strain for the 1B subgroup LMG 10829. The other four strain of this cluster presented the pattern of subgroup 1A (*n* = 2) and a different pattern to those described (*n* = 2). Cluster II (*n* = 6) corresponded to the four strains that showed a 16S rDNA-RFLP pattern similar to the one described for *A. butzleri* (Figueras et al., 2008) and two other strains with the patterns for the subgroups 1A and 1B. Cluster III, included the type strain of *A. cryaerophilus* LMG 24291^T^ and three field isolates from Chilean animals all belonging to the subgroup 1A, and Cluster IV comprised six strains, mostly from subgroup 1A (*n* = 5). Interestingly, strains recovered from human specimens belonged exclusively to Cluster I, suggesting potential host specificity because strains associated with farm animal abortions were present in the four clusters (Figure 1).

**Figure 2:**
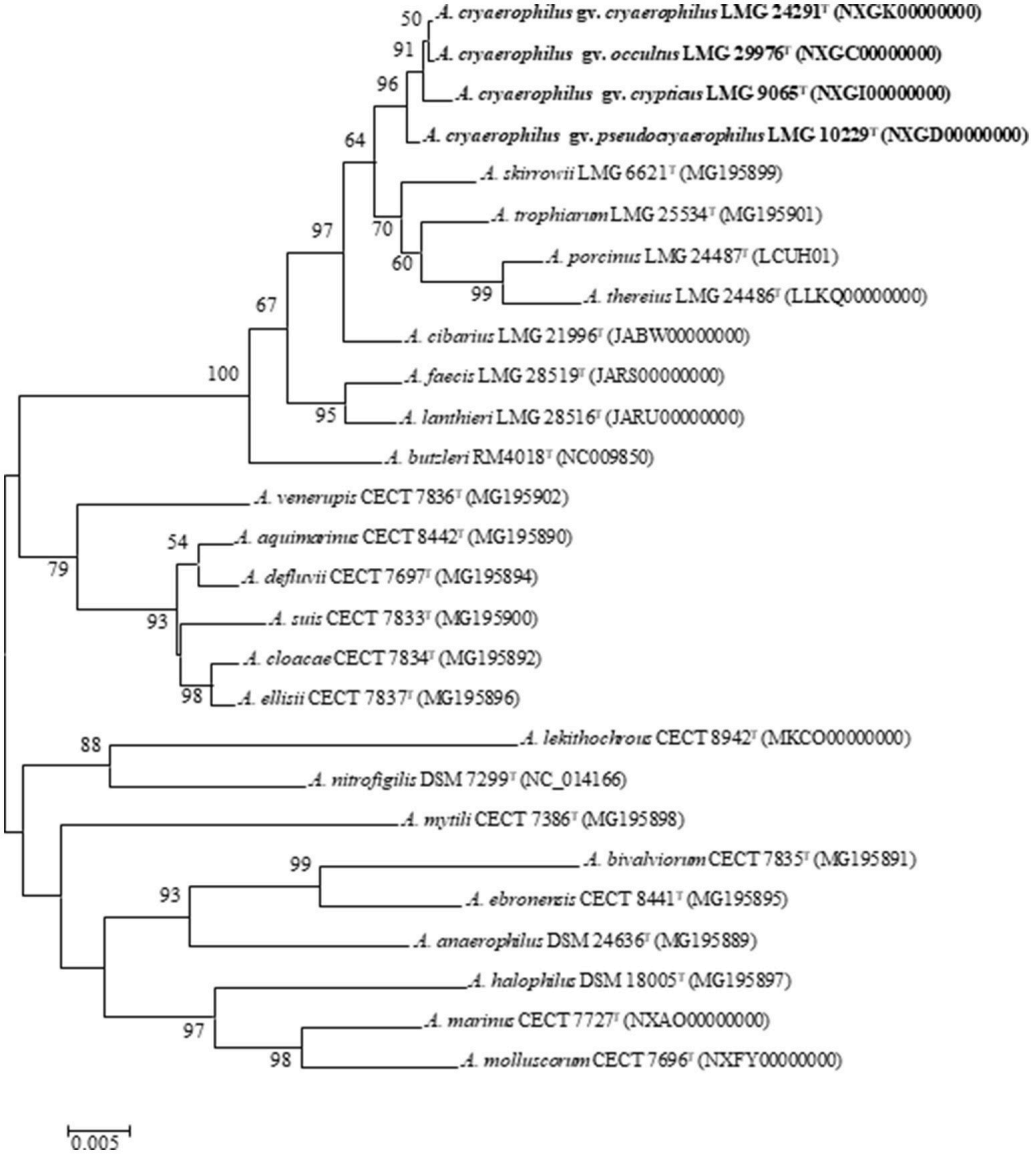
Neighbor joining tree based on 16S rRNA (1,496 bp) sequences showing the phylogenetic position of the representative strains of the four clusters of *A. cryaerophilus* within the genus *Arcobacter*. Bootstrap values (>50%) based on 1,000 replications are shown at the nodes of the tree. Bar, 5 substitutions per 1,000 bp.

A representative type strain was selected from each cluster (I–IV) for further analysis and for constructing a 16S rRNA gene phylogenetic tree (Figure 2). The tree showed that the four strains formed separated branches, strains LMG 24291^T^ and LMG 29976^T^ being the nearest ones. The percentage of similarity of the 16S rRNA gene between the type strains ranged from 99.5% between strains LMG 10229^T^ (Cluster I) and LMG 9065^T^ (Cluster II) to 99.9% between the original type strain of *A. cryaerophilus* LMG 24291^T^ (Cluster III) and the representative strain of Cluster IV (LMG 29976^T^). These results agree with what occurs between other species of *Arcobacter*, such as *A. ellisii* and *A. cloacae* (Figueras et al., 2011b; Levican et al., 2013b), where the 16S rRNA gene does not have enough resolution to differentiate the species. The phylogeny of the 23S rRNA gene (Supplementary Figure S1) and the one carried out with the concatenated sequences of the two rRNA genes (Supplementary Figure S2) presented the same topology shown with the 16S rRNA gene (Figure 2) and confirmed that the strains of Cluster III are more closely related to Cluster IV than to the other clusters.

### Genome analysis

**Figure 3:**
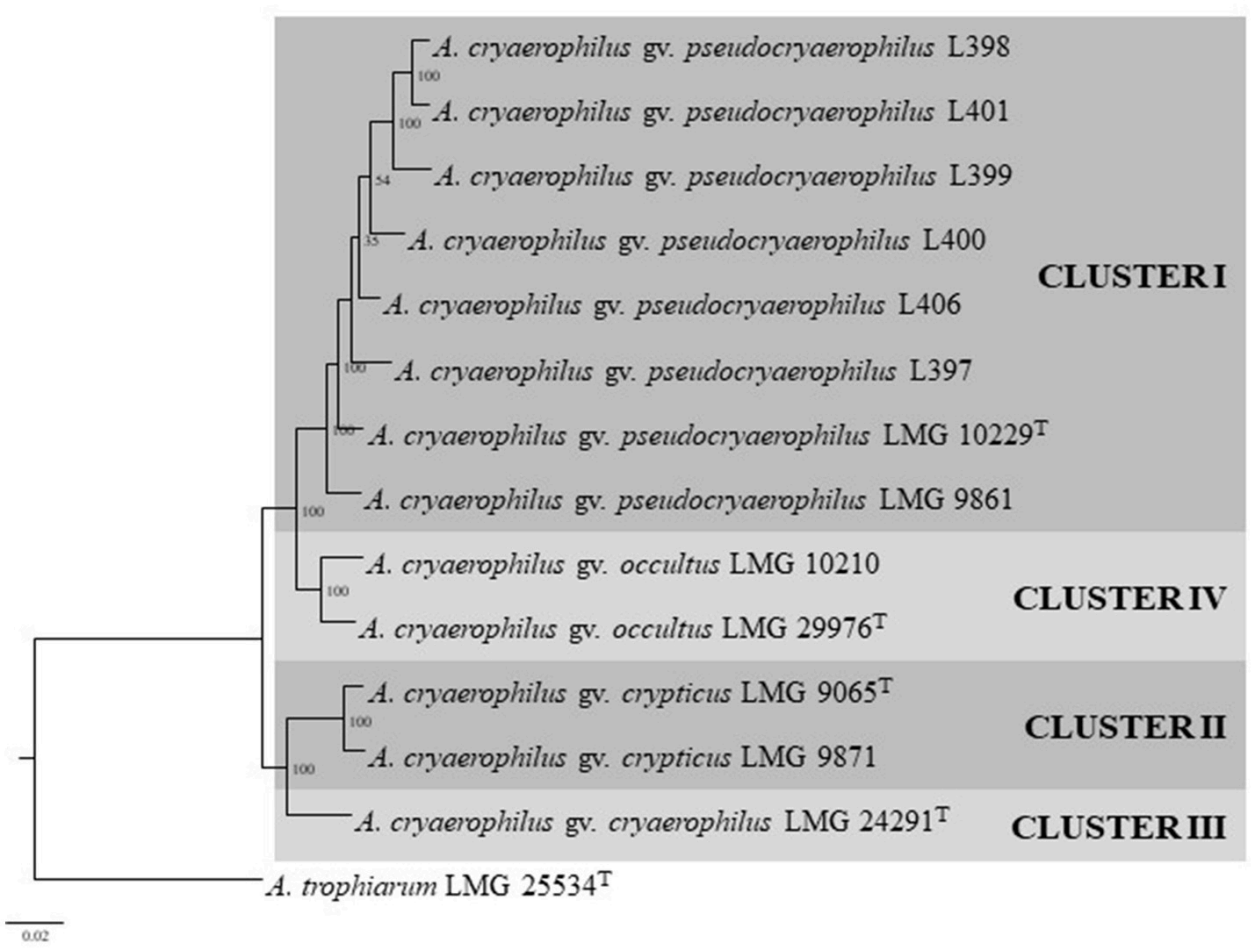
Maximum likelihood tree based on the concatenated sequences of 893 core protein sequences showing the distribution of the 13 genomes used in the same four clusters shown in Figure 1. Boostrap values based on 1,000 replications are shown at the nodes of the tree. Bar indicates 2 substitution per 100 aa.

The characteristics of the 13 compared genomes (8 representatives of Cluster I, two of clusters II and IV and one of Cluster III) are shown in Table 2. The quality of the genome sequences was in general in agreement with the minimal standards established for the use of genome data for taxonomical purposes, that embraces characteristics of the sequencing and assembly of the genomes like the depth of coverage, the value of N50 and the number of contigs (Chun et al., 2018). The exceptions were the genome sequence data of strains LMG 10229^T^, LMG 9871, and LMG 9861 that presented a depth of coverage lower than 50X proposed in the standards (Table 2). Globally, the genomic characteristics of the 13 compared genomes shown in Table 2 were very similar, with sizes that did not differ in more than 0.29 Mb, with a %mol G+C content ranging between 27.0 and 30.0% and with a number of coding sequences or CDS of around 2000 (Table 2). The G+C values were in agreement with those (24.6–31%) described in the recent emended description of the genus *Arcobacter* (Sasi Jyothsna et al., 2013). Table 3 shows the results from the calculated overall genome related taxonomical indices i.e., ANI and *is*DDH. For species delineation the generally accepted ANI and isDDH boundary values are 95–96 and 70%, respectively (Goris et al., 2007; Richter and Rossello-Mora, 2009; Meier-Kolthoff et al., 2013; Chun et al., 2018). However, for the genus *Arcobacter*, ANI values above 96% were the ones that better correlated with *is*DDH results above 70% in previous studies (Figueras et al., 2017; Pérez-Cataluña et al., 2018) in agreement with what happens in other genera (Beaz-Hidalgo et al., 2015; Figueras et al., 2017; Liu et al., 2017). The ANI values of the representative strains from each of the four different clusters were below the 96% cut-off indicating that the compared genomes belonged to different species, while the intra-cluster ANI values ranged from 96.6 to 98.6%. The *is*DDH results of <70% found between strains of the four clusters confirmed as the ANI results did that each cluster represented an independent species. The core genome phylogenetic tree inferred from 893 protein sequences of the 13 genomes obtained with PATRIC showed that the genomes also grouped into four different well-supported clusters with bootstraps of 100% (Figure 3). Interestingly, clusters IV and I formed a separate branch from clusters II and III. This indicates that the proteins of the genomes of clusters I and IV are more similar than the nucleotide sequences, and this was also in agreement with the higher values observed with ANI and *is*DDH for these two clusters.

**Table 2:** Characteristics of the 13 genomes from representative strains from each of the clusters.

**CLUSTERS**													
	**I**								**II**		**III**	**IV**	
**Features**	**LMG 10229^T^ (NXGD01^a^)**	**LMG 9861 (NXGJ01^a^)**	**L397 (LRUQ01^a^)**	**L398 (LRUR01^a^)**	**L399 (LRUS01^a^)**	**L400 (LRUT01^a^)**	**L401 (LRUU01^a^)**	**L406 (LRUV01^a^)**	**LMG9065^T^ (NXGI01^a^)**	**LMG9871 (NXGH01^a^)**	**LMG 24291^T^ (NXGK01^a^)**	**LMG 29976^T^ (NXGK01^a^)**	**LMG 10210 (NXGE01^a^)**
Deep Coverage	25X	37X	180X	129X	77X	106X	133X	190X	74X	19X	196X	187X	160X
Size (Mb)	2.06	2.02	2.31	2.03	2.10	2.20	2.17	2.02	2.05	2.08	2.05	2.19	2.26
Contigs	27	32	96	71	92	92	85	65	56	180	91	322	70
N50 (Kb)	199	109	56	64	54	54	58	64	138	38	54	241	355
G+C%	27.3	27.6	27.0	27.2	27.4	27.3	27.1	27.4	27.3	28.2	27.2	30.0	27.6
Genes (Total)	2,134	2,074	2,373	2,100	2,214	2,258	2,205	2,102	2,139	2,237	2,141	2,475	2,346
CDS (Coding)	2,071	2,000	2,246	2,002	2,100	2,138	2,117	2,020	2,070	2,136	2,081	2,288	2,255
Genes (RNA)	46	52	33	31	38	37	30	34	54	50	49	59	59
tRNAs	36	40	27	25	30	29	25	29	38	40	40	46	43
ncRNAs	2	3	3	2	3	2	2	2	2	2	3	3	3

a *Genome accession number; ncRNAs, non-coding RNAs*.

**Table 3:** Results of Average Nucleotide Identity (ANI) and *in silico* DNA-DNA hibridization (*is*DDH) between representative genomes of the four clusters.

**CLUSTER**													
	**I**								**II**		**III**	**IV**	
	**LMG 10229^T^**	**LMG 9861**	**L397**	**L398**	**L399**	**L400**	**L401**	**L406**	**LMG 9065^T^**	**LMG 9871**	**LMG 24291^T^**	**LMG 10210**	**LMG 29976^T^**
**Cluster I**													
**LMG 10229**^T^		74.7	74.3	72.3	73.5	73.5	71.9	75.7	50.3	50.4	50.1	63.7	62.0
**LMG 9861**	**97.2**		69.6	69.6	68.7	70.3	69.7	72.3	50.2	49.6	49.7	63.0	62.1
**L397**	**97.1**	**96.6**		73.5	75.3	78.4	71.9	82.1	49.3	49.0	49.2	60.1	59.9
**L398**	**96.9**	**96.6**	**97.1**		78.4	74.9	87.7	75.3	49.7	50.3	49.3	60.1	59.7
**L399**	**97.1**	**96.6**	**97.3**	**97.7**		75.3	79.0	75.8	49.8	49.5	49.5	60.2	59.2
**L400**	**97.1**	**96.7**	**97.7**	**97.2**	**97.3**		74.1	83.4	49.1	49.3	49.1	60.3	59.7
**L401**	**96.9**	**96.6**	**97.1**	**98.6**	**97.7**	**97.1**		74.6	49.9	49.9	50.5	60.7	59.5
**L406**	**97.3**	**96.9**	**98.1**	**97.3**	**97.4**	**98.2**	**97.2**		20.1	50.0	49.7	61.7	60.5
**Cluster II**													
**LMG 9065**^T^	**93.1**	**93.1**	**92.9**	**93.1**	**93.1**	**92.9**	**93.1**	**93.0**		81.4	56.1	51.1	51.7
**LMG 9871**	**93.1**	**93.1**	**92.9**	**93.1**	**93.0**	**92.9**	**93.1**	**93.0**	**98.1**		49.7	51.0	52.2
**Cluster III**													
**LMG 24291**^T^	**92.9**	**92.8**	**92.6**	**92.6**	**92.7**	**92.5**	**92.9**	**92.9**	**94.3**	**94.4**		52.3	54.4
**Cluster IV**													
**LMG 10210**	**95.7**	**95.8**	**95.2**	**95.2**	**95.3**	**95.3**	**95.2**	**95.5**	**93.5**	**93.4**	**93.4**		73.4
**LMG 29976**^T^	**95.2**	**95.5**	**95.1**	**94.9**	**94.9**	**95.1**	**94.9**	**95.1**	**93.5**	**93.5**	**93.9**	**97.1**	

*Values in bold in the lower triangle corresponds to ANI and in the upper triangle to isDDH*.

### Virulence and antibiotic resistance genes

Of the different methods and databases used for recognizing virulence factors (Victors, VFDB and PATRIC_VF) none of them were useful for recognizing virulence genes. There were only a few exceptions. The phospholipase C identified with the databases PATRIC_VF and Victors in the genome of the strain LMG 29976^T^ (Cluster IV). The enzyme UDP-N-acetylglucosamine 4-6 dehidratase involved in flagelline glycosylation and identified with the VFDB database in the genomes L397 and L399 (Cluster I). Finally, the Pspa protein (EC 2.3.1.41), essential for gluconeogenesis, identified using PATRIC_VF in the genome LMG 9871 of Cluster II (Table 4). However the BLASTn carried out for the detection of virulence genes showed the presence of different genes related with adhesion (*cj1349*), invasion (*ciaB* and *mviN*) and phospholipase activity (*pldA*) (Table 4). None of the genomes showed the *cadF, hecA* and *hecB* genes that encode a fibronectin binding protein, an adhesion protein and a factor for hemolysis activation, respectively. These results agree with those obtained for the genome of *A. thereius* LMG 24486^T^ (Rovetto et al., 2017). The *irgA* and *tlyA* genes that encode an iron-regulated outer membrane protein and a hemolysine, respectively, were the only ones found i.e. the gene *irgA* in the genomes L398 and L401 (Cluster I); the gene *tlyA* in LMG 24291^T^ (Cluster III) and LMG 9065^T^ (Cluster II). The phylogenetic analysis of the concatenated sequences of the four virulence genes present in all the genomes (*cj1349, mviN, pldA* and *ciaB*) formed the same four clusters (Supplementary Figure S3). However, the distribution of the clusters was similar to the one obtained with the core genome tree (Figure 3), where clusters I and IV formed a separated branch from clusters II and III.

**Table 4:** Antibiotic resistant genes and virulence factors.

**CLUSTERS**													
	**I**								**II**		**III**	**IV**	
	**1**	**2**	**3**	**4**	**5**	**6**	**7**	**8**	**9**	**10**	**11**	**12**	**13**
**ANTIBIOTIC RESISTANCE**													
**Multidrug efflux pumps**													
CmeABC system^a^	+	+	+	+	+	+	+	+	+	+	+	+	+
MFS Superfamily^a^	−	+	−	+	+	+	+	+	+	−	−	−	−
**Macrolids**													
MacAB−TolC^a^	+	+	+	+	+	+	+	+	+	+	+	+	+
**Quinolones**													
*gyrA* mutation	−	−	−	−	−	−	−	−	−	−	−	−	−
23S rRNA mutations	−	−	−	−	−	−	−	−	−	−	−	−	−
OqxB^b^	+	+	+	+	+	+	+	+	+	+	+	+	+
β**-lactamics**													
β-lactamase^a^	−	−	−	−	−	−	−	+^d^	−	−	−	−	−
**Colistin**													
Mcr−1^b^	+	−	+	+	+	+	+	+	+	−	+	−	+
Mcr−2^b^	+	−	+	+	+	+	+	+	+	−	+	−	+
Acriflavin resistance^a^	+	+	+	+	+	+	+	+	+	+	+	+	+
Streptomycin/Spectinomycin^a^	−	−	−	−	−	−	−	−	−	−	−	+	−
**VIRULENCE FACTORS**													
**Invasion**													
*ciaB*^c^	+	+	+	+	+	+	+	+	+	+	+	+	+
*mviN*^c^	+	+	+	+	+	+	+	+	+	+	+	+	+
**Adhesion**													
*cj1349*^c^	+	+	+	+	+	+	+	+	+	+	+	+	+
*cadF*^c^	−	−	−	−	−	−	−	−	−	−	−	−	−
**Filamentous hemmaglutinin**													
*hecA*^c^	−	−	−	−	−	−	−	−	−	−	−	−	−
**Hemolysis**													
*hecB*^c^	−	−	−	−	−	−	−	−	−	−	−	−	−
*tlyA*^c^	−	−	−	−	−	−	−	−	+	−	+	−	−
**Outer membarne protein**													
*irgA*^c^	−	−	−	+	−	−	+	−	−	−	−	−	−
**Phospholipase**													
*pldA*^c^	+	+	+	+	+	+	+	+	+	+	+	+^e^	+

*1, A. cryaerophilus gv. pseudocryaerophilus LMG 10229^T^; 2, A. cryaerophilus gv. pseudocryaerophilus LMG 9861; 3, A. cryaerophilus gv. pseudocryaerophilus L397; 4, A. cryaerophilus gv. pseudocryaerophilus L398; 5, A. cryaerophilus gv. pseudocryaerophilus L399; 6, A. cryaerophilus gv. pseudocryaerophilus L400; 7, A. cryaerophilus gv. pseudocryaerophilus L401; 8, A. cryaerophilus gv. pseudocryaerophilus L406; 9, A. cryaerophilus gv. crypticus LMG 9065^T^; 10, A. cryaerophilus gv. crypticus LMG 9871; 11, A. cryaerophilus gv. cryaerophilus LMG 24291^T^; 12, A. cryaerophilus gv. occultus LMG 29976^T^; 13, A. cryaerophilus gv. occultus LMG 10210*.

a *RAST/PATRIC results*,

b *ARG–ANNOT results*,

c *BLASTn of virulence genes results (See Supplementary Table S2)*,

d *β-lactamase class D*,

e *Phospholipase A and C*.

Regarding the presence of antibiotic resistant mechanisms, all the genomes showed the cmeABC multidrug efflux pump, the MacAB-TolC system for macrolide resistance, the *oxqB* gene related with quinolone resistance and genes related with the resistance to acriflavine. Resistance to colistin by the genes *mcr-1* and *mcr-2* were present in 85% of the genomes. The genome L406 was the only one that possessed a β-lactamase gene of class D. Resistance to β-lactamic compounds have been reported in other studies (Atabay and Aydin, 2001; Fera et al., 2003) and the same β-lactamase gene is present in the genome of *A. butleri* RM4018. However, this gene is absent in the genome of *A. thereius* LMG 24486^T^ (Rovetto et al., 2017). The genome LMG 29976 was the only one that presented genes for the resistance to streptomycin/spectomycin. The susceptibility of *A. cryaerophilus* to streptomycin has been previously demonstrated (Kabeya et al., 2004; Rahimi, 2014). However, this is the first report that show the presence of resistance genes to this antimicrobial compound. Mutations on the 23S rRNA (Ren et al., 2011) and the *gyrA* gene (Carattoli et al., 2002) for erythromycin and quinolone resistance were not detected, despite *gyrA* mutations have been found in some quinolone-resistant *A. cryaerophilus* strains (Abdelbaqi et al., 2007; Van den Abeele et al., 2016).

### Functional and phenotypic inference

**Figure 4:**
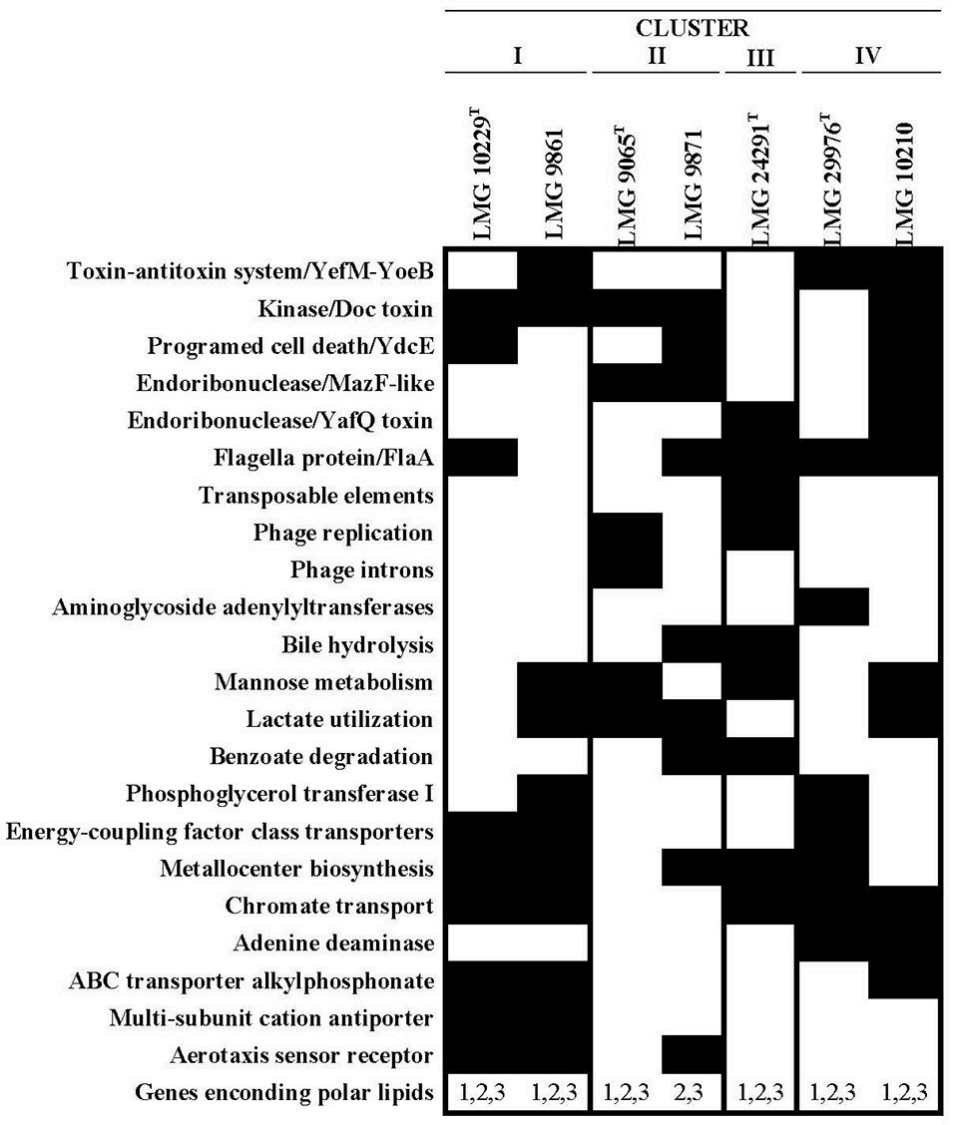
Function based comparison between the representative genomes of each cluster using RAST annotation results. Black squares represent de presence and white squares the absence of each subsystem/protein. 1-3 Polar lipids genes: 1. Phosphatidilglycerol phosphatase A (*pspA*); 2. phosphatidate cytidylyltransferase (*cdsA*); 3. phosphatidyl serine decarboxylase (*psd*). Genes *pspA* and *cdsA* are involved in the synthesis of phosphatidilglycerol (PG) and *psd* gene in the synthesis of phosphatidylethanolamine (PE).

Several subsystems where found to be characteristic of each Cluster on the basis of the functional-based comparison between the representative genomes (Figure 4). Cluster I genomes (LMG 10229^T^ and LMG 9861) carry specifically multi-subunit cation antiporters [Na(+) H(+) cation antiporter ABCDEFG] whose function includes sodium tolerance and pH homeostasis in an alkaline environment (Ito et al., 2017). Cluster II genomes (LMG 9065^T^ and LMG 9871) were the only ones that did not show the chromate transport protein ChrA, which confers resistance to chromate compounds present in the other studied genomes. Cluster III (LMG 24291^T^) was the only one that presented transposable elements as the putative transposase TniA and the Nucleotide Triphosphate binding protein TniB. Finally, the enzyme Adenosine deaminase (EC 3.5.4.4) involved in purine metabolism was only detected in Cluster IV genomes (LMG 29976^T^ and LMG 10210).

**Figure 5:**
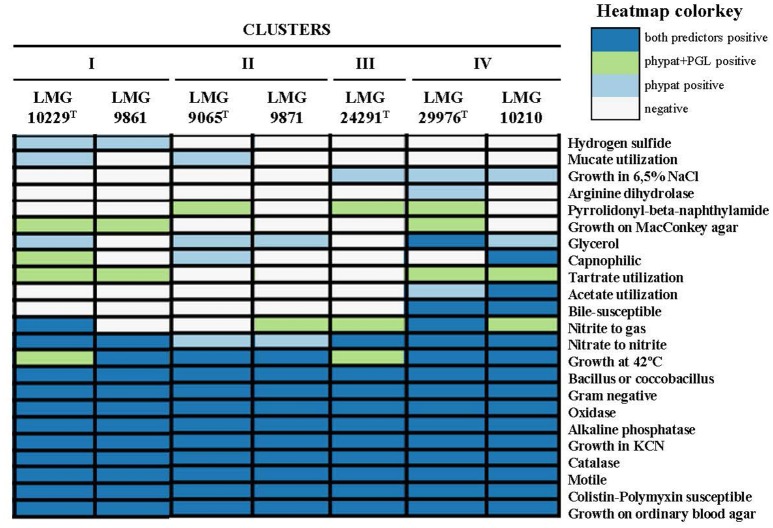
Phenotypic inference using Traitar software for the representative genomes of each cluster. The software uses two prediction models, the phypat model (predicts the presence/absence of proteins found in the phenotype of 234 bacterial species) and a combination of phypat+PGL models (uses the information of phypat combined with the information of the acquisition or loss of protein families and phenotypes through the evolution), to determine the phenotypic characteristics.

From the 67 phenotypic inferred traits analyzed with Traitar 11(16.4%) were found in all the analyzed genomes while 12 were only found in some of them (Figure 5). The genomes of Cluster I (LMG 10229^T^ and LMG 9861) were predicted to produce hydrogen sulfide while those of Cluster IV (LMG 29976^T^ and LMG 10210) showed acetate utilization and bile susceptibility. However, none of these characteristics have been observed when they have been tested in the laboratory on those strains. This might be due to the inability to reproduce the necessary conditions in the laboratory for the expression of these features. None of the other nine traits recognized in some genomes enabled us to differentiate between the IV Clusters.

### Phenotypical characterization

Table 5 shows the phenotypical results obtained from the strains of each of the four clusters. In agreement with what was found in previous studies where phenotypic test did not differentiate between subgroups 1A and 1B (Neill et al., 1985; Vandamme et al., 1992; On, 1996), none of the performed phenotypic tests enabled to clearly distinguish strains from each of the four phylogenetic clusters. Most of the tests gave variable results except for Cluster IV. However, this might be due to the small number of strains (*n* = 2) analyzed in this group. Considering these results, each of the three genetically recognized new species (clusters I, II, and IV) should be considered a different genomovar (gv.) of the species *A. cryaerophilus*. A genomovar is a well-delimited group of strains that correspond to a new species by genomic information but that cannot be phenotypically differentiated (Ursing et al., 1995). Cluster III represents the original species *A. cryaerophilus* because it embraces the type strain of the species. The value of the phenotypic characterization has already been questioned considering the lack of reproducibility of results between laboratories and some authors have suggested it is now time to base the description of new taxa on the genome sequence analysis (Moore et al., 2010; Sutcliffe, 2015). According to Sutcliffe (2015), phenotypic characterization is harder to evaluate nowadays than the genotype. Considering that genomic characterization is objective and reproducible, we agree with Sutcliffe (2015) that we should be able to define species on the basis of genetic characters like the ones evaluated in this study. This will favor the faster discover of the large number of taxa waiting to be described (Sutcliffe, 2015). However, this will require a modification of the Bacteriological Code, which we hope will happen in the near future.

**Table 5:** Phenotypic characteristics of the four clusters.

**Characteristics**	**1**	**2**	**3**	**4**
**Growth in/on**				
Air at 37°C	+	V(+)	+	−
Microaerobiosis at 37°C	+	V(+)	V(+)	−
Anaerobiosis at 37°C	V(+)	V(−)	V(−)	−
Air at 42°C	V(−)	−	−	−
2% (w/v) NaCl	V(+)	+	V(+)	+
3% NaCl	V(−)	−	−	−
4% (w/v) NaCl	−	−	−	−
1% bilis	+	V(+)	V(+)	+
1.5% bilis	+	+	V(+)	+
2% bilis	+	+	V(+)	+
1% (w/v) glycine	V(−)	−	−	−
0.1% sodium deoxycholate	V(+)	V(+)	V(+)	+
MacConkey	V(+)	+	V(+)	+
CdCl_2_	V(−)	V(−)	V(−)	V
**Resistance to**				
Cefoperazone (64 mg/L)	V(+)	+	+	+
**Enzyme activity**				
Catalase	+	+	+	+

*Taxa: 1, A. cryaerophilus gv. pseudocryaerophilus (n = 27) [Cluster I]; 2, A. cryaerophilus gv. crypticus (n = 5) [Cluster II]; 3, A. cryaerophilus gv. cryaerophilus(n = 3) [Cluster III]; 4, A. cryaerophilus gv. occultus (n = 2) [Cluster IV]. The specific responses for type strains were coincidental or expressed in brackets. Unless otherwise indicated: +, ≥95% strains positive; −, ≤ 11% strains positive; V, variable; (), main result of the strains; CO_2_ indicates microaerobic conditions*.

## Conclusion

The phylogenetic and genomic analysis showed that the strains of the species *A. cryaerophilus* represent four separated species. In addition, phenotypical and functional traits were in evidence for the genomes selected as representative of each cluster. Despite all the results, phenotypic characterization carried out at the laboratory showed a high inter- and intra-cluster variability that did not allow us to determine specific phenotypic characteristics or therefore to define the three uncovered clusters as three new species. Following current bacterial taxonomic rules, we will not be able to define these species until we find phenotypical characteristics that allow us to discriminate the three new species from each other and from the species *A. cryaerophilus*. Therefore, we describe them as four genomovars with the names “*A. cryaerophilus gv. pseudocryaerophilus*” (pseu.do.cry.a.e.ro'phi.lus. Gr. adj. *pseudês* false, N.L. masc. adj. *cryaerophilus* specific epithet of an *Arcobacter* species; N.L. masc. adj. *pseudocryaerophilus* false *cryaerophilus;* Cluster I = LMG 10229^T^), “*A. cryaerophilus gv. crypticus*” (cryp'ti.cus. L. masc. adj. *Crypticus* hidden; Cluster II = LMG 9065^T^), *A. cryaerophilus gv. cryaerophilus* (Cluster III = LMG 24291^T^) and “*A. cryaerophilus gv. occultus*” (oc.cul′tus. L. adj. *occultus* occulted, hidden; Cluster IV = LMG 29976^T^). Unfortunately, the phenotype derived from the genome could not be reproduced in the laboratory, either. This might be due to the inability to mimic *in vitro* the conditions for the expression of these pathways or characteristics. The phenotypic characterization limits a proper description and it might be considered an important shortcoming in the genomic era in which all the molecular and genomic data leave no doubts about the existence of four different species among the investigated *A. cryaerophilus* strains.

## Authors contributions

LC and MF: designed the work; LC and OS: carried out the phylogenetic analysis; VL and AP-C: carried out the phenotypic characterization of the strains; AP-C: carried out the genome sequencing and analysis; LC, MF, and AP-C: wrote the paper.

### Conflict of interest statement

The authors declare that the research was conducted in the absence of any commercial or financial relationships that could be construed as a potential conflict of interest.

## Abbreviations

LMG: Laboratorium voor Microbiologie, Universiteit Gent, Belgium Culture Collection
MLPA: Multilocus Phylogenetic Analysis
ANI: Average Nucleotide Identity
*is*DDH: *in silico* DNA-DNA hybridization.
