# Supplementary material for: A Polyphasic and Taxogenomic Evaluation Uncovers Arcobacter cryaerophilus as a Species Complex That Embraces Four Genomovars
Source: Front Microbiol. 2018 Apr 27;9:805. doi: 10.3389/fmicb.2018.00805 (PMC5934430; doi:10.3389/fmicb.2018.00805)
Supplement: Supplementary file 1 [file Presentation_1.PDF]

A polyphasic and taxogenomic evaluation uncovers *Arcobacter cryaerophilus* as a species complex that embraces four genomovars

Alba Pérez-Cataluña[1], Luis Collado[2], Oscar Salgado[2,3], Violeta Lefiñanco[2] and María José Figueras[1*]

[1]Unit of Microbiology, Department of Basic Health Sciences, Faculty of Medicine and Health Sciences, IISPV, University Rovira i Virgili, Reus, Spain.

[2]Institute of Biochemistry and Microbiology, Faculty of Sciences, Universidad Austral de Chile, Valdivia, Chile.

[3]Laboratory of Microbial Ecology of Extreme Systems, Department of Molecular Genetics and Microbiology, Pontificia Universidad Católica de Chile, Santiago, Chile.

SUPPLEMENTARY MATERIAL

Supplelemtary Figures S1-S4

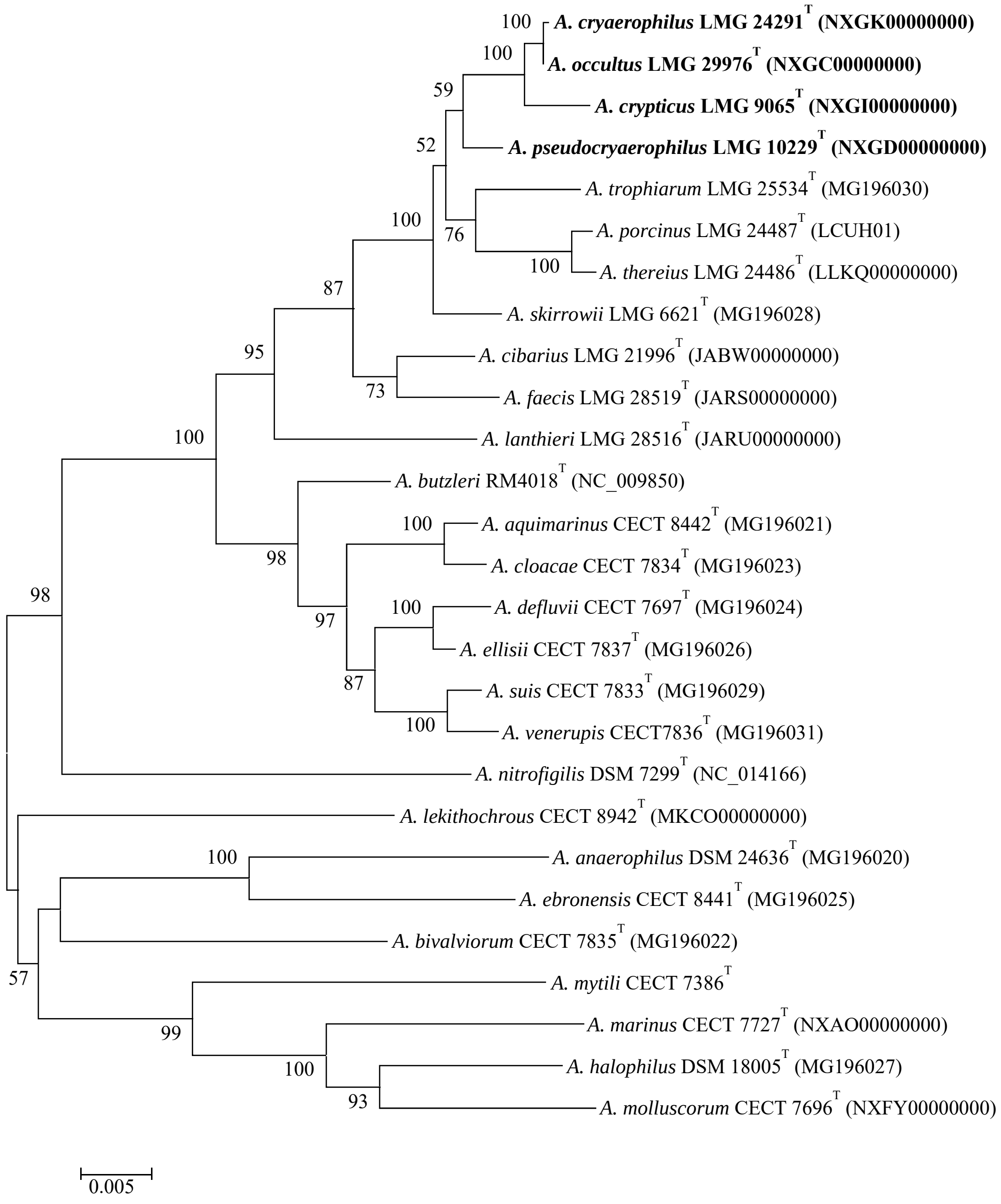

Supplementary Figure S1. Neighbour joining tree based on 23S rRNA (2857 bp) sequences showing the phylogenetic position of the three new species in relation with *A. cryaerophilus* and within the genus *Arcobacter*. Bootstrap values (>50%) based on 1000 replications are shown at the nodes of the tree. Bar, 5 substitutions per 1000 bp.

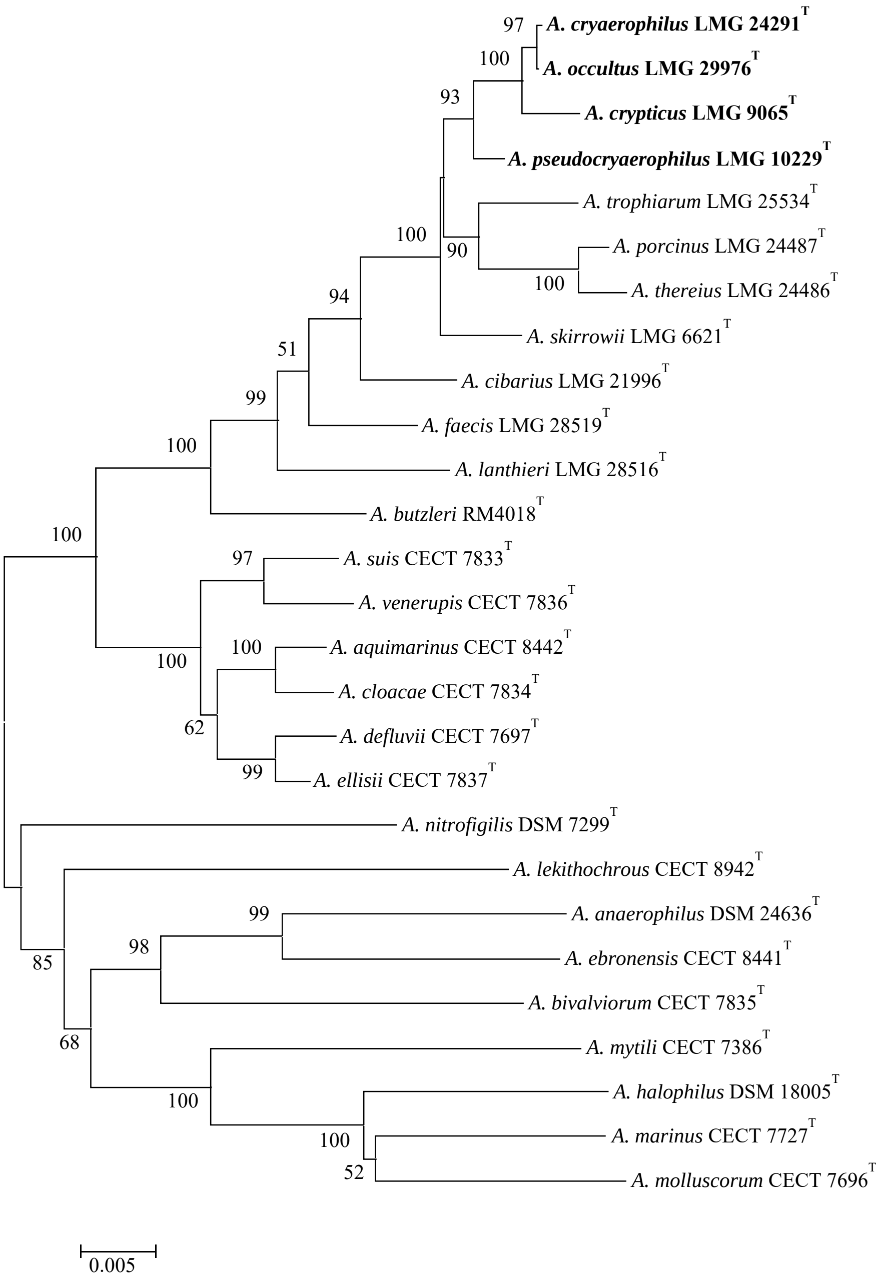

Supplementary Figure S2. Neighbour joining tree based on the concatenated sequences of the 16S and 23S rRNA genes (4353 bp) sequences showing the phylogenetic position of the three new species within the genus *Arcobacter* and in relation with *A. cryaerophilus*. Bootstrap values (>50%) based on 1000 replications are shown at the nodes of the tree. Bar, 5 substitutions per 1000 bp.

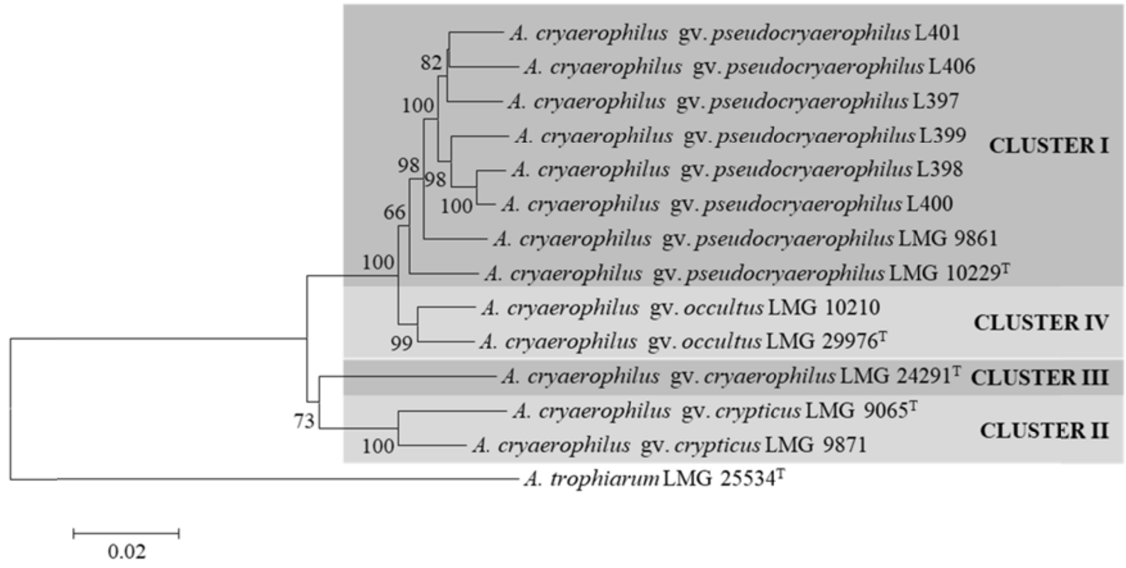

Supplementary Figure S3. Neighbor-joining tree based on the concatenated sequences of the four virulence genes *cj1349*, *mviN*, *pldA* and *ciaB* (5386 bp) found in the 13 genomes. Notice the same four clusters observed in Fig. 1. Bootstrap values (>50%) based on 1000 replications are shown at the nodes of the tree. Bar indicates 2 substitutions per 100 bp.

Supplementary Figure S4. MLPA alignment

LMG24291T	ATTTGTTGCTGATTTAAATAAAGATGCAGCTTTGTGTGATGTTATGCACTTTAGTGATAAAGTAGATGGTGTAGAAGT	[78]
LMG10241	........................AA....AC.A.....A........T..............T..............	[78]
LMG10229	........................GA....AC.A.....A........T..............T........T.....	[78]
LMG9861	........................GA....AC.A.....A........T..............T........T.....	[78]
LMG9065	....	[78]
LMG9871	....	[78]
LMG 10210	....	[78]
LMG9865	....	[78]
L397	........................GA....AC.A.....A..A.....T..............T........T.....	[78]
L398	........................GA....AC.A.....G........T........C.....T........T.....	[78]
L399	........................GA....AC.A.....A........T..............T..C.....T.....	[78]
L400	........................GA....AC.A.....A........T..............T........T.....	[78]
L401	........................GA....AC.A.....A........T..............T..............	[78]
L406	........................GA....AC.A.....A........T..............T..............	[78]
14PHA	........................GA....AC.A.....A........T..............T..............	[78]
20PHF	........................GA....AC.A.....A........T..............T..............	[78]
284/1	........................GA....AC.A.....A........T..............T..............	[78]
8122333	........................GA....AC.A.....A........T..............T..............	[78]
8749401	........................GA....AC.A.....A........T..............T..C.....T.....	[78]
8756347	........................GA....AC.A.....A........T..............T........T.....	[78]
AB3A	........................GA....AC.A.....A........T..............T........T.....	[78]
AB74A	........................GA....AC.A.....A........T..............T..............	[78]
AL 20-1	....	[78]
AO2A	........................AA....AC.A.....A........T..............T..............	[78]
BUF3	........................GA....AC.A.....A........T..............T..............	[78]
CV-152	..........................T...	[78]
CV-2101	....	[78]
EMU-3	....	[78]
F196	........................GA....AC.A.....A........T..............T..............	[78]
FE 7	....	[78]
FEBU4	........................GA....AC.A.....A........T..............T........T.....	[78]
HHS 118A	........................GA....AC.A.....A........T..............T..............	[78]
HHS 133	........................GA....AC.A.....G........T..............T..............	[78]
HHS 188A	........................GA....AC.A.....A........T..............T..............	[78]
HHS 191A	........................GA....AC.A.....A........T..............T..............	[78]
HHS 205A	........................GA....AC.A.....A........T..............T..............	[78]
LMG 10829	........................GA....AC.A.....A........T..............T..............	[78]
LMG 9863	....	[78]
MC 2-2	........................GA....AC.A.....A........T..............T..............	[78]
ME 15-4	....	[78]
MICV 42-1	........................GA....AC.A.....A........T..............T........T.....	[78]
NAV 12-2	........................GA....AC.A..C..G........T..............T..............	[78]
NAV 15-1	.................................A..	[78]
NB14A	........................AA....AC.A.....A........T..............T..............	[78]
RW15-1	....	[78]
RW17-4	....	[78]
RW25-5	........................GA....AC.A.....A........T..............T..............	[78]
RW33-8	........................GA....AC.A.....A........T..............T..C.....T.....	[78]
RW45-3	.................a.a..	[78]
UF1T	........................GA....AC.A.....A........T..............T........T.....	[78]
UF2T	........................GA....AC.A.....A........T........C.....T........T.....	[78]
UPER3	........................GA....AC.A.....A........T..............T........T.....	[78]
A. trophiarum LMG25534T	G........A...A.G........GA....AC.T..............T..............T..............	[78]

LMG24291T	TGACATTGCAATGATGTATAACGATACATATATTGAAAAAACACTATCTTTTGTAAATAATATTAGAACAATTGATGG	[156]
LMG10241	...T.....T........C...........C...........T...................................	[156]
LMG10229	.........T....................C...........T...................................	[156]
LMG9861	.........T....................C...........T...................................	[156]
LMG9065	.........T................................T...................................	[156]
LMG9871	.........T................................T...................................	[156]
LMG 10210	....	[156]
LMG9865	...T.....T...G........	[156]
L397	...T.....T........C.......................T...................................	[156]
L398	.........T........C...........C...........T...................................	[156]
L399	...T.....T........C...........C...........T...................................	[156]
L400	...T.....T........C.......................T...................................	[156]
L401	...T.....T........C...........C...........T.............................A.....	[156]
L406	...T.....T........C...........C...........T...................................	[156]
14PHA	...T.....T........C...........C...........T.............................A.....	[156]
20PHF	...T.....T........C...........C...........T...................................	[156]
284/1	...T.....T........C...........C...........T...................................	[156]
8122333	...T.....T........C...........C...........T...................................	[156]
8749401	...T.....T........C...........C...........T...................................	[156]
8756347	...T.....T........C...........C...........T...................................	[156]
AB3A	.........T........C.......................T...................................	[156]
AB74A	...T.....T........C...........C...........T...................................	[156]
AL 20-1	.........T................................T...................................	[156]
AO2A	...T.....T........C...........C...........T...................................	[156]
BUF3	...T.....T........C...........C...........T...................................	[156]
CV-152	....	[156]
CV-2101	....	[156]
EMU-3	....	[156]
F196	...T.....T....................C...........T...................................	[156]
FE 7	.........T................................T...................................	[156]
FEBU4	...T.....T........C.......................T...................................	[156]
HHS 118A	...T.....T........C...........C...........T...................................	[156]
HHS 133	.........T....................C...........T...................................	[156]
HHS 188A	...T.....T........C...........C...........T...................................	[156]
HHS 191A	...T.....T....................C...........T...................................	[156]
HHS 205A	...T.....T........C...........C...........T...................................	[156]
LMG 10829	...T.....T........C...........C...........T...................................	[156]
LMG 9863	.........T................................T...................................	[156]
MC 2-2	...T.....T........C...........C...........T...................................	[156]
ME 15-4	.........T................................T...................................	[156]
MICV 42-1	...T.....T........C.......................T...................................	[156]
NAV 12-2	...T.....T........C...........C...........T...................................	[156]
NAV 15-1	.........T................................T..........................G........	[156]
NB14A	...T.....T........C...........C...........T...................................	[156]
RW15-1	.........T................................T..........................T........	[156]
RW17-4	.........T................................T..........................T........	[156]
RW25-5	...T.....T....................C...........T...................................	[156]
RW33-8	...T.....T........C...........C...........T...................................	[156]
RW45-3	.........T................................T..........................G........	[156]
UF1T	...T.....T........C.......................T................................C..	[156]
UF2T	.........T........C...........C...........T...................................	[156]
UPER3	...T.....T........C...........C...........T...................................	[156]
A. trophiarum LMG25534T	A..T.....TT.A.............................T...AG...............AC.......A.....	[156]

LMG24291T	AGGAACACATGAAGCTGGTTTTAAAGCAGGACTTACAAGAAGTATTTCTAAATATTTAAGTGAAAATGCAGCTGCAAG	[234]
LMG10241	T.....T....................T.................C................................	[234]
LMG10229	T.....T....................T.................C................................	[234]
LMG9861	T..C................................	[234]
LMG9065	T.....T...........A....................................C......................	[234]
LMG9871	T.....T...........A...	[234]
LMG 10210	....	[234]
LMG9865	....	[234]
L397	T.....T....................T.................C................................	[234]
L398	T.....T.......................G..............C................................	[234]
L399	T.....T....................T.................C................................	[234]
L400	T.....T....................T.................C................................	[234]
L401	T.....T....................T.................C................................	[234]
L406	T.....T....................T.................C................................	[234]
14PHA	T.....T....................T.................C..........................A..T..	[234]
20PHF	T.....T....................T..	[234]
284/1	T.....T....................T.................C................................	[234]
8122333	T.....T....................T.................C................................	[234]
8749401	T.....T....................T..	[234]
8756347	T.....T......................................C..........................A..T..	[234]
AB3A	T.....T..........................A...........C................................	[234]
AB74A	T.....T......................................C..........................A..T..	[234]
AL 20-1	T.....T...........A...	[234]
AO2A	T.....T.......................T...A..T..	[234]
BUF3	T.....T....................T.................C................................	[234]
CV-152	....	[234]
CV-2101	....	[234]
EMU-3	....	[234]
F196	T.....T....................T.................C................................	[234]
FE 7	T.....T...........A...	[234]
FEBU4	T.....T......................................C................................	[234]
HHS 118A	T.....T....................T..	[234]
HHS 133	T.....T......................................C................................	[234]
HHS 188A	T.....T....................T.................C..........................A..T..	[234]
HHS 191A	T.....T......................................C................................	[234]
HHS 205A	T.....T......................................C................................	[234]
LMG 10829	T.....T....................T..	[234]
LMG 9863	T.....T...........A....................................C......................	[234]
MC 2-2	T.....T....................T.................C..........................A..T..	[234]
ME 15-4	T.................A...	[234]
MICV 42-1	T.....T......................................C................................	[234]
NAV 12-2	T.....T......................................C................................	[234]
NAV 15-1	T..G.....G....................	[234]
NB14A	T.....T.......................T...A..T..	[234]
RW15-1	T.....G..C......................................G..G.....G....................	[234]
RW17-4	T.....G..C......................................G..G.....G....................	[234]
RW25-5	T.....T....................T.................C................................	[234]
RW33-8	T.....T....................T..	[234]
RW45-3	T.....G..C......................................G..G.....G....................	[234]
UF1T	T.....T......................................C..........................A..T..	[234]
UF2T	C.....T......................................C................................	[234]
UPER3	T.....T....................T.................C................................	[234]
A. trophiarum LMG25534T	T..............G..G.....................TC......A.......................A...C.	[234]

LMG24291T	AGAGAAAGATGCAAAAATAACTGGCGATGATGTAAGAGAAGGTTTAATAGCAGTTGTTTCAGTAAAAGTTCCAGAACC	[312]
LMG10241	...A....................A.................AC.T..T...........T.................	[312]
LMG10229	...A....................A.................AC.T..T...........T.................	[312]
LMG9861	...A....................A.................AC.T..T...........T.................	[312]
LMG9065	...A....................A...T.................	[312]
LMG9871	........................A...T.................	[312]
LMG 10210	....	[312]
LMG9865	........................T...	[312]
L397	...A....................A.................AC.T..T...........T.................	[312]
L398	...A....................A..................C.T..T...........T.................	[312]
L399	...A....................A..................C.T..T...........T.................	[312]
L400	...A....................A.................GC.T..T...........T.................	[312]
L401	...A....................A..................C.T..T...........T.................	[312]
L406	...A....................A.................AC.T..T...........T.................	[312]
14PHA	...A....................A.................AC.T..T...........T.................	[312]
20PHF	...A....................G..................C.T..T...........T.................	[312]
284/1	...A....................A.................AC.T..T...........T.................	[312]
8122333	...A....................A.................AC.T..T...........T.................	[312]
8749401	...A....................A.................AC.T..T...........T.................	[312]
8756347	...A....................A..................C.T..T...........T.................	[312]
AB3A	...A....................A.................GC.T..T...........T.................	[312]
AB74A	...A....................A.................AC.T..T...........T.................	[312]
AL 20-1	........................A....................................T.................	[312]
AO2A	...A....................A..................C.T..T...........T........A........	[312]
BUF3	...A....................A..............G..GC.T..T...........T.................	[312]
CV-152	....	[312]
CV-2101	....	[312]
EMU-3	....	[312]
F196	...A....................A.................AC.T..T...........T.................	[312]
FE 7	........................A....................................T.................	[312]
FEBU4	...A....................A.................GC.T..T...........T.................	[312]
HHS 118A	...A....................A..................C.T..T...........T.................	[312]
HHS 133	...A..............T.....A.................GC.T..T...........T.................	[312]
HHS 188A	...A....................A.................GC.T..T...........T.................	[312]
HHS 191A	...A....................A.................AC.T..T...........T.................	[312]
HHS 205A	...A..............T.....A.................GC.T..T...........T.................	[312]
LMG 10829	...A....................A..................C.T..T...........T.................	[312]
LMG 9863	...A....................A....................................T.................	[312]
MC 2-2	...A....................A..................C.T..T...........T.................	[312]
ME 15-4	........................A....................................T.................	[312]
MICV 42-1	...A....................A.................GC.T..T...........T.................	[312]
NAV 12-2	...A....................A..................C.T..T...........T.................	[312]
NAV 15-1	........................T...	[312]
NB14A	...A....................A..................C.T..T...........T........A........	[312]
RW15-1	........................T...	[312]
RW17-4	........................T...	[312]
RW25-5	...A....................A..................C.T..T...........T.................	[312]
RW33-8	...A....................A..................C.T..T...........T.................	[312]
RW45-3	........................T..............G........T..T..........................	[312]
UF1T	...A....................A.................GC.T..T...........T.................	[312]
UF2T	...A....................A..................C.T.............T.................	[312]
UPER3	...A....................A..................C.T..T...........T.................	[312]
A. trophiarum LMG25534T	G..A.......T.........A..A.................GC.T..T...........T...........T.....	[312]

LMG24291T	TCAATTTGAAGGACAAACAAAAGGAAAATTAGGAAGTTCTTATGTAAGACCTATTGCCCAAAAACTAACAGGTGATAA	[390]
LMG10241	A...........T......................................A.....A........T...A.......	[390]
LMG10229	............T.......................C..............A.....A........T...A.......	[390]
LMG9861	............T......................................A.....A........T...A.......	[390]
LMG9065	...G................................C..............A.....T.............C......	[390]
LMG9871	...G....................T...........C..............A.....T....................	[390]
LMG 10210	....	[390]
LMG9865	....................................C.....................T......T..........C..	[390]
L397	A...........T......................................A.....A........T...A.......	[390]
L398	............T......................................A.....A........T...A.......	[390]
L399	A...........T......................................A.....A........T...A.......	[390]
L400	A...........T......................................A.....A........T...A.......	[390]
L401	............T......................................A.....A........T...A.......	[390]
L406	A...........T......................................A.....A........T...A.......	[390]
14PHA	A...........T......................................A.....A........T...A.......	[390]
20PHF	A...........T......................................A.....A........T...A.......	[390]
284/1	A...........T......................................A.....A........T...A.......	[390]
8122333	A...........T......................................A.....A........T...A.......	[390]
8749401	A...........T...........G..........................A.....A........T...A.......	[390]
8756347	............T......................................A.....A........T...A.......	[390]
AB3A	............T......................................A.....A........T...A.......	[390]
AB74A	A...........T......................................A.....A........T...A.......	[390]
AL 20-1	...G....................T...........C..............A.....T....................	[390]
AO2A	A..G........T......................................A.....A........T...A.......	[390]
BUF3	A...........T......................................A.....A........T...A.......	[390]
CV-152	....	[390]
CV-2101	....	[390]
EMU-3	....	[390]
F196	A...........T...........G..........................A.....A........T...A.......	[390]
FE 7	...G....................T...........C..............A.....T....................	[390]
FEBU4	............T......................................A.....A........T...A.......	[390]
HHS 118A	............T......................................A.....A........T...A.......	[390]
HHS 133	............T......................................A.....A........T...A.......	[390]
HHS 188A	A...........T......................................A.....A........T...A.......	[390]
HHS 191A	............T......................................A.....A........T...A.......	[390]
HHS 205A	............T.......................C..............A.....A........T...A.......	[390]
LMG 10829	............T......................................A.....A........T...A.......	[390]
LMG 9863	...G....................T...........C.....................T......T......C......	[390]
MC 2-2	............T......................................A.....A........T...A.......	[390]
ME 15-4	...G....................T...........C..............A.....T....................	[390]
MICV 42-1	............T......................................A.....A........T...A.......	[390]
NAV 12-2	............T......................................A.....A........T...A.......	[390]
NAV 15-1	....................................C.....................T......T..........C..	[390]
NB14A	A..G........T......................................A.....A........T...A.......	[390]
RW15-1	....................................C.....................T......T..........C..	[390]
RW17-4	....................................C.....................T......T..........C..	[390]
RW25-5	............T......................................A.....A........T...A.......	[390]
RW33-8	A...........T...........G..........................A.....A........T...A.......	[390]
RW45-3	....................................C.....................T......T..........C..	[390]
UF1T	............T.......................C..............A.....A........T...A.......	[390]
UF2T	............T......................................A.....A........T...A.......	[390]
UPER3	A...........T...........G..........................A.....A........T...A.......	[390]
A. trophiarum LMG25534T	A...........T.......................C..............A.....A......T....T........	[390]

LMG24291T	C T T A G A T A A A T A T T T T G A A G A A A A T C C T A C A C A A G C A A G A G C T G T T A T G G A A A A A T C T T T A A T G G C A G C A C G A G G A C G	[468]
LMG10241	T . . . . C . . . . A . . . . . T . . . . A . . . . G . . . . T . . . . . T . . T A .	[468]
LMG10229	T . . . . A . . . . . T . . . . A . . . . G . . . . A . . . . . A .	[468]
LMG9861	T . . . . A . . T . . T . . . . A . . . . G . . . . T . . . . . T . . T A .	[468]
LMG9065	. . . . G . . . . . G . . . . A .	[468]
LMG9871	. . . . G . . . . A . . . . . A .	[468]
LMG 10210	. . . .	[468]
LMG9865	. . . . G . . . . A . . . . . A .	[468]
L397	T . . . . C . . . . A . . . . . T . . . . A . . . . G . . . . T . . . . . T . . T A .	[468]
L398	T . . . . A . . . . . T . . . . A . . . . G . . . . T . . . . . T . . T A .	[468]
L399	T . . . . C . . . . A . . . . . T . . . . A . . A . . . . G . . . . T . . . . . T . . T A .	[468]
L400	T . . . . C . . . . A . . . . . T . . . . A . . . . G . . . . T . . . . . T . . T A .	[468]
L401	T . . . . A . . . . . T . . . . A . . . . G . . . . T . . . . . T . . T A .	[468]
L406	T . . . . C . . . . A . . . . . T . . . . A . . . . G . . . . T . . . A . . . . T A .	[468]
14PHA	T . . . . A . . T . . T . . . . A . . . . G . . . . T . . . A . . . . T A .	[468]
20PHF	T . . . . C . . . . A . . . . . T . . . . A . . A . . . . G . . . . T . . . . . T . . T A .	[468]
284/1	T . . . . C . . . . A . . . . . T . . . . A . . . . G . . . . T . . . A . . . . T A .	[468]
8122333	T . . . . A . . . . . T . . . . A . . . . G . . . . T . . . A . . . . . A .	[468]
8749401	T . . . . C . . . . A . . . . . T . . . . A . . A . . . . G . . . . T . . . . . T . . T A .	[468]
8756347	T . . . . A . . T . . T . . . . A . . . . G . . . . T . . . . T A .	[468]
AB3A	T . . . . A . . T . . T . . . . A . . . . G . . . . G . . . A . . . . . A .	[468]
AB74A	T . . . . C . . . . A . . . . . T . . . C . T . . . . . A . . . . G . . . . T . . . . . T . . T A .	[468]
AL 20-1	. . . . G . . . . A . . . . . A .	[468]
AO2A	T . . . . C . . . . A . . . . . T . . . . A . . . . G . . . . G . . . A . . . . . A .	[468]
BUF3	T . . . . A . . T . . T . . . . A . . . . G . . . . T . . . . . T . . T A .	[468]
CV-152	. . . .	[468]
CV-2101	. . . .	[468]
EMU-3	. . . .	[468]
F196	T . . . . C . . . . A . . . . . T . . . . A . . A . . . . G . . . . T . . . A . . . . T A .	[468]
FE 7	. . . . G . . . . A . . . . . A .	[468]
FEBU4	T . . . . A . . T . . T . . . . A . . . . G . . . . T . . . . . T . . T A .	[468]
HHS 118A	T . . . . C . . . . A . . . . . T . . . . A . . . . G . . . . T . . . A . . . . T A .	[468]
HHS 133	T . . . . A . . . . . T . . . . A . . . . G . . . . T . . . . . T . . T A .	[468]
HHS 188A	T . . . . C . . . . A . . . . . T . . . . A . . . . G . . . . T . . . . . T . . T A .	[468]
HHS 191A	T . . . . C . . . . A . . . . . T . . . C . T . . A . . A . . . . G . . . . T . . . A . . . . . A .	[468]
HHS 205A	T . . . . A . . T . . T . . . . A . . . . G . . . . T . . . . . T . . T A .	[468]
LMG 10829	T . . . . C . . . . A . . . . . T . . . . A . . . . G . . . . T . . . A . . . . T A .	[468]
LMG 9863	. . . . G . . . . . G . . . . A .	[468]
MC 2-2	T . . . . C . . . . A . . . . . T . . . C . T . . A . . A . . . . G . . . . G . . . A . . . . . A .	[468]
ME 15-4	. . . . G . . . . A . . . . . A .	[468]
MICV 42-1	T . . . . A . . T . . T . . . . A . . . . G . . . . T . . . . . T . . T A .	[468]
NAV 12-2	T . . . . A . . . . . T . . . . A . . . . G . . . . T . . . . . T . . T A .	[468]
NAV 15-1	. . . .	[468]
NB14A	T . . . . C . . . . A . . . . . T . . . . A . . . . G . . . . G . . . A . . . . . A .	[468]
RW15-1	. . . . G . . . . A . . . . . A .	[468]
RW17-4	. . . . G . . . . A . . . . . A .	[468]
RW25-5	T . . . . C . . . . A . . . . . T . . . . A . . . . G . . . . T . . . . . T . . T A .	[468]
RW33-8	T . . . . C . . . . A . . T . . T . . . C . T . . . . . A . . . . G . . . . T . . . A . . . . T A .	[468]
RW45-3	. . . . G . . . . A . . . . . A .	[468]
UF1T	T . . . . A . . . . . T . . . . A . . . . G . . . . G . . . A . . . . . A .	[468]
UF2T	T . . . . A . . T . . T . . . . A . . . . G . . . . T . . . . . T . . T A .	[468]
UPER3	T . . . . A . . T . . T . . . . A . . . . G . . . . T . . . . . T . . T A .	[468]
A. trophiarum LMG25534T	T C . . . . G . . . . A T G . . . . C . T . . . . A . . . . . A .	[468]

LMG24291T	TGAAGCAGCTAAAAAAGCAAGAGAACTAACTAGAAAAAAAAGATTCTATGAGTGTTGGAACACTTCCTGGAAAACTTGC	[546]
LMG10241	A.....T..................T.................C...........A.......................	[546]
LMG10229	A.....T..................T.................C...........A.......................	[546]
LMG9861	A.....T..................T.................C...........A.......................	[546]
LMG9065	A.....T..	[546]
LMG9871	A.....T..	[546]
LMG 10210	....	[546]
LMG9865	A.....T....................................C...................................	[546]
L397	A.....T..................T.................C...........A.......................	[546]
L398	A.....T.................GT.................C...........A.......................	[546]
L399	A.....T..................T.................C...................................	[546]
L400	A.....T..................T.................C...........A.......................	[546]
L401	A.....T..................T.................C...........A.......................	[546]
L406	A.....T..................T.................C...................................	[546]
14PHA	A.....T..................T.................C...........A.......................	[546]
20PHF	A.....T..................T.................C...................................	[546]
284/1	A.....T..................T.................C...................................	[546]
8122333	A.....T..................T.................C...................................	[546]
8749401	A.....T..................T.................C...........A.......................	[546]
8756347	A.....T...........G......T.................C...........A.......................	[546]
AB3A	A.....T.................GT.................C...........A.......................	[546]
AB74A	A.....T..................T.G...............C...................................	[546]
AL 20-1	A.....T..	[546]
AO2A	A.....T.................GT.................C...................................	[546]
BUF3	A.....T..................T.................C...................................	[546]
CV-152	....	[546]
CV-2101	....	[546]
EMU-3	....	[546]
F196	A.....T..................T.................C...........A.......................	[546]
FE 7	A.....T..	[546]
FEBU4	A.....T..................T.................C...........A.......................	[546]
HHS 118A	A.....T..................T.................C...................................	[546]
HHS 133	A.....T..................T.................C...........A.......................	[546]
HHS 188A	A.....T..................T.................C...........A.......................	[546]
HHS 191A	A.....T..................T.................C...................................	[546]
HHS 205A	A.....T..................T.................C...........A.......................	[546]
LMG 10829	A.....T..................T.................C...................................	[546]
LMG 9863	A.....T..	[546]
MC 2-2	A.....T..................T.................C...................................	[546]
ME 15-4	A.....T..	[546]
MICV 42-1	A.....T..................T.................C...........A.......................	[546]
NAV 12-2	A.....T..C...............T.................C...................................	[546]
NAV 15-1	....	[546]
NB14A	A.....T.................GT.................C...................................	[546]
RW15-1	A.....T....................................C...................................	[546]
RW17-4	A.....T....................................C...................................	[546]
RW25-5	A.....T..................T.................C...........A.......................	[546]
RW33-8	A.....T..................T.................C...................................	[546]
RW45-3	A.....T.................GT.................C...................................	[546]
UF1T	A.....T..................T.................C...........A.......................	[546]
UF2T	A.....T..................T.................C...................................	[546]
UPER3	A.....T..................T.................C...........A.......................	[546]
A. trophiarum LMG25534T	A.....T..................T...........................A.A...........A..T...T.A..	[546]

LMG24291T	TGATTGTCAAAGTAAAGATCCAACTATAAGAGAGTTATATCTAGTGGAAGGGGACTCTGCGGGAGGTGGAGAGGGTGG	[624]
LMG10241	....T........T.................	[624]
LMG10229	............C................................A........T.....A.................	[624]
LMG9861	............C................................A..............A.................	[624]
LMG9065	A...T..A..T........T........	[624]
LMG9871	A...T..A..T........T........	[624]
LMG 10210	....T....................A.....	[624]
LMG9865	....T..TAG...T...........A.....	[624]
L397	....T........T.................	[624]
L398	............C...........A.T.A...........T....T..G..T........T.................	[624]
L399	....T........T.................	[624]
L400	........................A.T.A...........T....T..G..T........A.................	[624]
L401	........................A.T.A.....C.G...T....T..G..T........A.................	[624]
L406	........................A.T.A.....C.G...T....T..G..T........A.................	[624]
14PHA	............C................................A..............T.................	[624]
20PHF	........................A.T.A.....C.G...T....T..G..T........A.................	[624]
284/1	....T........T.................	[624]
8122333	........................A.T.A.....C.G...T....T..G..T........A.................	[624]
8749401	........................A.T.A.....C.G...T....T..G..T........A.................	[624]
8756347	..........................T........................T........T.................	[624]
AB3A	............C................................A........T.....A.................	[624]
AB74A	........................A.T.A.....C.G...T....T..G..T........A.................	[624]
AL 20-1	A...T..A..T........T........	[624]
AO2A	A.......................A.T.A.....C.G...T....T..G..T........A.................	[624]
BUF3	..................C.....A.T.A.....C.G...T....T..G..T........A.................	[624]
CV-152	....T..........................	[624]
CV-2101	....T..........................	[624]
EMU-3	....T..........................	[624]
F196	....T.....A.................	[624]
FE 7	A...T..A..T........T........	[624]
FEBU4	............C...........A.T.A.....C.G...T....T..G..T........A.................	[624]
HHS 118A	........................A.T.A.....C.G...T....T..G..T........A.................	[624]
HHS 133	....T.....A.................	[624]
HHS 188A	....T........T.................	[624]
HHS 191A	..................C.....A.T.A.....C.G...T....T..G..T........A.................	[624]
HHS 205A	............C................................A..............A.................	[624]
LMG 10829	........................A.T.A.....C.G...T....T..G..T........A.................	[624]
LMG 9863	A...T..A..T........T........	[624]
MC 2-2	........................A.T.A.....C.G...T....T..G..T........A.................	[624]
ME 15-4	A...T..A..T........T........	[624]
MICV 42-1	............C...........A.T.A.....C.G...T....T..G..T........A.................	[624]
NAV 12-2	........................A.T.A.....C.G...T....T..G..T........A.................	[624]
NAV 15-1	....T....................A.....	[624]
NB14A	A.......................A.T.A.....C.G...T....T..G..T........A.................	[624]
RW15-1	....T..TAG...T...........A.....	[624]
RW17-4	....T..TAG...T...........A.....	[624]
RW25-5	..................C.....A.T.A.....C.G...T....T..G..T........A.................	[624]
RW33-8	....T........T.................	[624]
RW45-3	....T..TAG...T...........A.....	[624]
UF1T	........................A.T.A.....C.G...T....T..G..T........A.................	[624]
UF2T	....T.................	[624]
UPER3	....T.....A.................	[624]
A. trophiarum LMG25534T	A.......................A...A...................G..T..T.....A...........A.....	[624]

LMG24291T	T C T T G T T A A A G A A A G A G C G G G A T T T G A A G T A A G G G A C G T T C A T C C A A C T C A C T A T G G A A G A A T T T G T C C A G T T G A G A C	[702]
LMG10241	. T . A . . A . . . . . G . . . . . A . . . . G	[702]
LMG10229	. T . A . . A . . . . . G . . . . . A . . . . G . . . . C	[702]
LMG9861	. T . A . . A . . . . . G . . . . . A	[702]
LMG9065	. T . A . . A . . . . T . . . . A . . T . . . . . C . . . . A . .	[702]
LMG9871	. T . A . . A . . . . . G . . . . . A . . . . A . . T . . . . . C . . . . A . .	[702]
LMG 10210	. T . A . . A . . . . . G . . . . . A . . . . C . . . . A . .	[702]
LMG9865	. T . A . . A . . . . . G . . . . . A . . . . C . . . . A . .	[702]
L397	. T . A . . A . . . . . G . . . . . A . . . . G . . . . C	[702]
L398	. T . A . . A . . . . . G . . . . . A . . . . G . . . . C . . . . A . .	[702]
L399	. T . A . . A . . . . . G . . . . . A . . . . G . . . . C . . . . A . .	[702]
L400	. T . A . . A . . . . . G . . . . . A . . . . G . . . . C	[702]
L401	. . . . A . . . . C	[702]
L406	. T . A . . A . . . . . G . . . . . A . . . . G	[702]
14PHA	. T . A . . A . . . . . G . . G . . A . . . . G . . . . C	[702]
20PHF	. T . A . . A . . . . . G . . . . . A . . . . G . . . . C	[702]
284/1	. T . A . . A . . . . . G . . . . . A . . . . G . . . . C	[702]
8122333	. T . A . . A . . . . . G . . . . . A . . . . G . . . . C	[702]
8749401	. T . A . . A . . . . . G . . . . . A . . . . G . . . . C	[702]
8756347	. T . A . . A . . . . . G . . . . . A . . . . G	[702]
AB3A	. T . A . . A . . . . . G . . . . . A . . . . C	[702]
AB74A	. T . A . . A . . . . . G . . . . . A . . . . G	[702]
AL 20-1	. T . A . . A . . . . . G . . . . . A . . . . A . . T . . . . . C . . . . A . .	[702]
AO2A	. T . A . . A . . . . . G . . . . . A . . . . G	[702]
BUF3	. T . A . . A . . . . . G . . . . . A . . . . G	[702]
CV-152	. . . . T	[702]
CV-2101	. . . .	[702]
EMU-3	. . . .	[702]
F196	. T . A . . A . . . . . G . . . . . A . . . . G . . . . C	[702]
FE 7	. T . A . . A . . . . A . . G . . . . A . . T . . . . . C . . . . A . .	[702]
FEBU4	. T . A . . A . . . . . G . . . . . A . . . . G . . . . C	[702]
HHS 118A	. T . A . . A . . . . . G . . . . . A . . . . G	[702]
HHS 133	. T . A . . A . . . . . G . . . . . A	[702]
HHS 188A	. T . A . . A . . . . . G . . . . . A . . . . G	[702]
HHS 191A	. T . A . . A . . . . . G . . . . . A . . . . G . . . . C	[702]
HHS 205A	. T . A . . A . . . . . G . . . . . A	[702]
LMG 10829	. T . A . . A . . . . . G . . . . . A . . . . G	[702]
LMG 9863	. T . A . . A . . . . . G . . . . . A . . . . A . . T . . . . . C . . . . A . .	[702]
MC 2-2	. T . A . . A . . . . . G . . . . . A . . . . G . . . . C	[702]
ME 15-4	. T . A . . A . . . . A . . G . . . . A . . T . . . . . C . . . . A . .	[702]
MICV 42-1	. T . A . . A . . . . . G . . . . . A . . . . C	[702]
NAV 12-2	. . . . A . . . . G . . . . C	[702]
NAV 15-1	. T . A . . A . . . . . G . . . . . A . . . . C . . . . A . .	[702]
NB14A	. T . A . . A . . . . . G . . . . . A . . . . G	[702]
RW15-1	. T . G . . A . . . . . G . . . . . A . . . . C . . . . A . .	[702]
RW17-4	. T . A . . A . . . . . G . . . . . A . . . . C . . . . A . .	[702]
RW25-5	. T . A . . A . . . . . G . . . . . A . . . . G . . . . C	[702]
RW33-8	. T . A . . A . . . . . G . . . . . A . . . . G	[702]
RW45-3	. T . A . . A . . . . . G . . . . . A . . . . C . . . . A . .	[702]
UF1T	. T . A . . A . . . . . G . . . . . A . . . . G . . . . C	[702]
UF2T	. T . A . . A . . . . . G . . . . . A . . . . G C . . . . T . . . . T	[702]
UPER3	. T . A . . A . . . . . G . . . . . A . . . . G . . . . C	[702]
A. trophiarum LMG25534T	. . . . G . . . . T . . . . A . . T . . . . . C . . . . C . . . . . T . . . . . A . .	[702]

LMG24291T	T C C A G A G G G T C A A A A T A T C G G T C T T A T A A A T A C T C T T T C A A C T T A T T C A A A A G T A A A T G A G C T A G G A T T T A T T G A A G C	[780]
LMG10241	. . . . T	[780]
LMG10229	. . . .	[780]
LMG9861	. . . . T	[780]
LMG9065	. . . . T . . . . T . . . . T	[780]
LMG9871	. . . . T . . . . T	[780]
LMG 10210	. . . . T	[780]
LMG9865	. . . . T	[780]
L397	. . . . T	[780]
L398	. . . . T	[780]
L399	. . . . T	[780]
L400	. . . . T	[780]
L401	. . . . T	[780]
L406	. . . . T	[780]
14PHA	. . . . T	[780]
20PHF	. . . . T	[780]
284/1	. . . . T	[780]
8122333	. . . . T	[780]
8749401	. . . . T	[780]
8756347	. . . . T	[780]
AB3A	. . . . A T	[780]
AB74A	. . . . T	[780]
AL 20-1	. . . . T . . . . T	[780]
AO2A	. . . . T	[780]
BUF3	. . . . T	[780]
CV-152	. . . .	[780]
CV-2101	. . . .	[780]
EMU-3	. . . .	[780]
F196	. . . . T	[780]
FE 7	. . . . T . . . . T	[780]
FEBU4	. . . . T	[780]
HHS 118A	. . . . T	[780]
HHS 133	. . . . A T	[780]
HHS 188A	. . . . T . . . . T	[780]
HHS 191A	. . . . T	[780]
HHS 205A	. . . . A T	[780]
LMG 10829	. . . . T	[780]
LMG 9863	. . . . T . . . . T	[780]
MC 2-2	. . . . T	[780]
ME 15-4	. . . . T . . . . T . . . . T	[780]
MICV 42-1	. . . . A T	[780]
NAV 12-2	. . . . T	[780]
NAV 15-1	. . . . T	[780]
NB14A	. . . . T	[780]
RW15-1	. . . . T	[780]
RW17-4	. . . . T	[780]
RW25-5	. . . . T	[780]
RW33-8	. . . . T	[780]
RW45-3	. . . . T	[780]
UF1T	. . . . T	[780]
UF2T	. . . . T	[780]
UPER3	. . . . A . . . . T	[780]
A. trophiarum LMG25534T	. . . . G . . . . . C . . . . C	[780]

LMG24291T	TCCTTATAAAAAAGTAGTTGATGGTGTTGTTACAAATGAAATTTCATACTACACTGCAACTCAAGAAGAGGGTCTTGT	[858]
LMG10241	....A.....	[858]
LMG10229	....A.....	[858]
LMG9861	....A.....	[858]
LMG9065	...........................A....................T.............................	[858]
LMG9871	....T.............................	[858]
LMG 10210	....	[858]
LMG9865	....	[858]
L397	....T....................A.....	[858]
L398	....T........T..............A.....	[858]
L399	....T........T..............A.....	[858]
L400	....G.....	[858]
L401	....T.................A..A.....	[858]
L406	....A.....	[858]
14PHA	....A.....	[858]
20PHF	....A.....	[858]
284/1	....A.....	[858]
8122333	....A.....	[858]
8749401	....A.....	[858]
8756347	....A.....	[858]
AB3A	....A.....	[858]
AB74A	....A.....	[858]
AL 20-1	....T.............................	[858]
AO2A	....A.....	[858]
BUF3	A...A.....	[858]
CV-152	....	[858]
CV-2101	....	[858]
EMU-3	....	[858]
F196	...............G..A.....	[858]
FE 7	....T.............................	[858]
FEBU4	.................................T......................................A.....	[858]
HHS 118A	....T..T..A.................A.....	[858]
HHS 133	....T..T..A.................A.....	[858]
HHS 188A	....A.....	[858]
HHS 191A	....A.....	[858]
HHS 205A	....	[858]
LMG 10829	....T....................A.....	[858]
LMG 9863	....T.............................	[858]
MC 2-2	....A.....	[858]
ME 15-4	....T..T..........................	[858]
MICV 42-1	....A.....	[858]
NAV 12-2	....A..A.....	[858]
NAV 15-1	....	[858]
NB14A	....A.....	[858]
RW15-1	....	[858]
RW17-4	....	[858]
RW25-5	....A.....	[858]
RW33-8	....T..T..A.................A.....	[858]
RW45-3	....	[858]
UF1T	....A.....	[858]
UF2T	....T..T..A.................A.....	[858]
UPER3	....A.....	[858]
A. trophiarum LMG25534T	...............T.........A.............G..C.....T..T..A...........G..A........	[858]

```
LMG24291T                  AATTGCTCCTGGTTCAACAAAACTTGATGAAAATGGAAAAATTATTGAACCACTTGTAGAAGTAAGATTAAATGGTGA [936]
LMG10241                   ......................G.............................T.AA.T.....T...C.T........ [936]
LMG10229                   ......A...............G.............................T.AA.C.....T...C.T........ [936]
LMG9861                    ......A...............G.............................T.AA.C.....T...C.T........ [936]
LMG9065                    ...............T................................G............................. [936]
LMG9871                    ...............T................................G............................. [936]
LMG 10210                  .........A..A...................................G............................. [936]
LMG9865                    ............G...................................G..........................C.. [936]
L397                       ......................G.............................T.AA.T.....T...C.T........ [936]
L398                       ......................G.............................T.AA.C.....T...C.T........ [936]
L399                       ......................G.............................T.AA.C.....T...C.T........ [936]
L400                       ......................G.............................T.AA.T.....T...C.T........ [936]
L401                       ......................G.............T...............T.AA.C.....T...C.T........ [936]
L406                       ......................G.............................T.AA.T.....T...C.T........ [936]
14PHA                      ......................G.............................T.AA.T.....T...C.T........ [936]
20PHF                      ......................G.............................T.AA.T.....T...C.T........ [936]
284/1                      ......................G.............................T.AA.T.....T...C.T........ [936]
8122333                    ......................G.............................T.AA.T.....T...C.T........ [936]
8749401                    ......................G.............................T.AA.T.....T...C.T........ [936]
8756347                    ......................G.............................T.AA.T.....T...C.T........ [936]
AB3A                       ......A...............G.............................T.AA.C.....T...C.T........ [936]
AB74A                      ......................G.............................T.AA.T.....T...C.T........ [936]
AL 20-1                    ...............T................................G............................. [936]
AO2A                       ......................G.............................T.AA.T.....T...C.T........ [936]
BUF3                       ......................G.............................T.AA.T.....T...C.T........ [936]
CV-152                     .............................................................................. [936]
CV-2101                    ...........................................................................C.. [936]
EMU-3                      ...........................................................................C.. [936]
F196                       ......................G.............................T.AA.T.....T...C.T........ [936]
FE 7                       ...............T.............................................................. [936]
FEBU4                      ......................G.............................T.AA.C.....T...C.T........ [936]
HHS 118A                   ......................G.............................T.AA.T.....T...C.T........ [936]
HHS 133                    ......A...............G.............................T.AA.C.....T...C.T........ [936]
HHS 188A                   ......................G.............................T.AA.T.....T...C.T........ [936]
HHS 191A                   ......................G.............................T.AA.T.....T...C.T........ [936]
HHS 205A                   ......A...............G.............................T.AA.C.....T...C.T........ [936]
LMG 10829                  ......................G.............................T.AA.T.....T...C.T........ [936]
LMG 9863                   ...............T................................G............................. [936]
MC 2-2                     ......................G.............................T.AA.T.....T...C.T........ [936]
ME 15-4                    ...............T................................G............................. [936]
MICV 42-1                  ......A...............G.............................T.AA.C.....T...C.T........ [936]
NAV 12-2                   ......................G.............T...............T.AA.C.....T...C.T........ [936]
NAV 15-1                   ............G...................................G............................. [936]
NB14A                      ......................G.............................T.AA.T.....T...C.T........ [936]
RW15-1                     ............G...................................G............................. [936]
RW17-4                     ............G...................................G..........................C.. [936]
RW25-5                     ......................G.............................T.AA.T.....T...C.T........ [936]
RW33-8                     ......................G.............................T.AA.T.....T...C.T........ [936]
RW45-3                     ............G...................................G..........................C.. [936]
UF1T                       ......................G.............................T.AA.T.....T...C.T........ [936]
UF2T                       ......................G.............................T.AA.T.....T...C.T........ [936]
UPER3                      ......................G.............................T.AA.T.....T...C.T........ [936]
A. trophiarum LMG25534T    ...C........AAGT....................G.....C........G.....T.................A.. [936]
```

LMG24291T	A A T C A T T T T A A T G G A T A A A A A T A A A G T T G A T T T A A T A G A T A T A T C A T C T C A A A T G G T A A T G G G G G T T G C G G C T T C T T T	[1014]
LMG10241	. . . T C . . . . G . . . . A . . . . T . . . . . A . . C	[1014]
LMG10229	. . . T . . . . . G . . . . T . . . . . T . . C	[1014]
LMG9861	. . . T . . . . . G . . . . A . . . . T . . . . . A . . C	[1014]
LMG9065	. . . .	[1014]
LMG9871	. . . .	[1014]
LMG 10210	. . . . T . . . . . T . . C	[1014]
LMG9865	. . . . T	[1014]
L397	. . . T . . . . . G . . . . A . . . . T . . . . . A . . C	[1014]
L398	. . . T C . . . . G . . . . A . . . . T . . . . . T	[1014]
L399	. . . T C . . . . G . . . . A . . . . T . . . . . T	[1014]
L400	. . . T . . . . . G . . . . A . . . . T . . . . . A . . C	[1014]
L401	. . . T C . . . . G . . . . A . . . . T . . . . . A . . C	[1014]
L406	. . . T . . . . . G . . . . A . . . . T . . . . . A . . C	[1014]
14PHA	. . . T . . . . . G . . . . A . . . . T . . . . . A . . C	[1014]
20PHF	. . . T . . . . . G . . . . A . . . . T . . . . . A . . C	[1014]
284/1	. . . T . . . . . G . . . . G . . . . A . . . . T . . . . . A . . C	[1014]
8122333	. . . T . . . . . G . . . . A . . . . T . . . . . A . . C	[1014]
8749401	. . . T . . . . . G . . . . A . . . . T . . . . . A . . C	[1014]
8756347	. . . T . . . . . G . . . . A . . . . T . . . . . A . . C	[1014]
AB3A	. . . T . . . . . G . . . . A . . . . T . . . . . A . . C	[1014]
AB74A	. . . T . . . . . G . . . . G . . . . A . . . . T . . . . . A . . C	[1014]
AL 20-1	. . . .	[1014]
AO2A	. . . T . . . . . G . . . . A . . . . T . . . . . A . . C	[1014]
BUF3	. . . T . . . . . G . . . . A . . . . T . . . . . A . . C	[1014]
CV-152	. . . .	[1014]
CV-2101	. . . .	[1014]
EMU-3	. . . .	[1014]
F196	. . . T . . . . . G . . . . A . . . . T . . . . . A . . C	[1014]
FE 7	. . . .	[1014]
FEBU4	. . . T . . . . . G . . . . A . . . . T . . . . . A . . C	[1014]
HHS 118A	. . . T . . . . . G . . . . A . . . . T . . . . . A . . C	[1014]
HHS 133	. . . T . . . . . G . . . . A . . . . T . . . . . A . . C	[1014]
HHS 188A	. . . T . . . . . G . . . . A . . . . T . . . . . A . . C	[1014]
HHS 191A	. . . T . . . . . G . . . . A . . . . T . . . . . A . . C	[1014]
HHS 205A	. . . T . . . . . G . . . . A . . . . T . . . . . A . . C	[1014]
LMG 10829	. . . T . . . . . G . . . . A . . . . T . . . . . A . . C	[1014]
LMG 9863	. . . .	[1014]
MC 2-2	. . . T . . . . . G . . . . A . . . . T . . . . . A . . C	[1014]
ME 15-4	. . . .	[1014]
MICV 42-1	. . . T . . . . . G . . . . A . . . . T . . . . . A . . C	[1014]
NAV 12-2	. . . T C . . . . G . . . . A . . . . T . . . . . A . . C	[1014]
NAV 15-1	. . . . T . . . . . T . . C	[1014]
NB14A	. . . T . . . . . G . . . . A . . . . T . . . . . A . . C	[1014]
RW15-1	. . . . T . . . . . T . . C	[1014]
RW17-4	. . . . T	[1014]
RW25-5	. . . T . . . . . G . . . . A . . . . T . . . . . A . . C	[1014]
RW33-8	. . . T . . . . . G . . . . A . . . . T . . . . . A . . C	[1014]
RW45-3	. . . . T . . . . . T . . C	[1014]
UF1T	. . . T C . . . . G . . . . A . . . . T . . . . . A . . C	[1014]
UF2T	. . . T C . . . . G . . . . A . . . . T . . . . . A . . C	[1014]
UPER3	. . . T . . . . . G . . . . A . . . . T . . . . . A . . C	[1014]
A. trophiarum LMG25534T	. . . . C . . . . C . . G . . . . T . . . . T . . . . . A . . . . . T . . . . . . C .	[1014]

```
LMG24291T                  A A T T C C A T T T T T A G A A C A C A A C G A T G C A A A T A G A G C A T T A A T G G G T T C A A A T A T G A T G A G A C A A G C T G T T C C A T T G A T [1092]
LMG10241                   . . . . . . . . . . . . . . . G . . . . . T . . . . . . . . . . . . . . . . . . . . . . . . . . . . . . . . . . . . . . . . . . . . . . . . . . . . . . [1092]
LMG10229                   . . . . . . . . . . . . . . . G . . . . . T . . . . . . . . . . . . . . . . . . . . . . . . . . . . . . . . . . . . . . . . . . . . . . . . . . . . . . [1092]
LMG9861                    . . . . . . . . . . . . . . . G . . . . . T . . . . . . . . . . . . . . . . . . . . . . . . . . . . . . . . . . . . . . . . . . . . . . . . . . . . . . [1092]
LMG9065                    . . . . . . . . . C . . . . . . . . T . . T . . . . . . . . . . . . . . . . . . . . . . . . . . . . . . . . . . . . . . . . . . . . . . . . . . . . . . [1092]
LMG9871                    . . . . . . . . . C . . . . . G . . . . . . . . . . . . . . . . . . . . . . . . . . . . . . . . . . . . . . . . . . . . . . . . . . . . . . . . . . . . [1092]
LMG 10210                  . . . . . . . . . C . . . . . . . . . . . . . . . . . . . . . . . . . . . . . . . . . . . . . . . . . . . . . . . . . . . . . . . . . . . . . . . . . . [1092]
LMG9865                    . . . . . . . . . . . . . . . . . . . . . . . . . . . . . . . . . . . . . . . . . . . . . . . . . . . . . . . . . . . . . . . . . . . . . . . . . . . . [1092]
L397                       . . . . . . . . . . . . . . . G . . . . . T . . . . . . . . . . . . . . . . . . . . . . . . . . . . . . . . . . . . . . . . . . . . . . . . . . . . . . [1092]
L398                       . . . . . . . . . . . . . . . G . . . . . T . . . . . . . . . . . . . . . . . . . . . . . . . . . . . . . . . . . . . . . . . . . . . . . . . . . . . . [1092]
L399                       . . . . . . . . . . . . . . . G . . . . . T . . . . . . . . . . . . . . . . . . . . . . . . . . . . . . . . . . . . . . . . . . . . . . . . . . . . . . [1092]
L400                       . . . . . . . . . . . . . . . G . . . . . T . . . . . . . . . . . . . . . . . . . . . . . . . . . . . . . . . . . . . . . . . . . . . . . . . . . . . . [1092]
L401                       . . . . . . . . . . . . . . . G . . . . . T . . . . . . . . . . . . . . . . . . . . . . . . . . . . . . . . . . . . . . . . . . . . . . . . . . T . . . . . [1092]
L406                       . . . . . . . . . . . . . . . G . . T . . T . . . . . . . . . . . . . . . . . . . . . . . . . . . . . . . . . . . . . . . . . . . . . . . . . . . . . . [1092]
14PHA                      . . . . . . . . . . . . . . . G . . T . . T . . . . . . . . . . . . . . . . . . . . . . . . . . . . . . . . . . . . . . . . . . . . . . . . . . . . . . [1092]
20PHF                      . . . . . . . . . . . . . . . G . . . . . T . . . . . . . . . . . . . . . . . . . . . . . . . . . . . . . . . . . . . . . . . . . . . . . . . . . . . . [1092]
284/1                      . . . . . . . . . . . . . . . G . . . . . T . . . . . . . . . . . . . . . . . . . . . . . . . . . . . . . . . . . . . . . . . . . . . . . . . . . . . . [1092]
8122333                    . . . . . . . . . . . . . . . G . . . . . T . . . . . . . . . . . . . . . . . . . . . . . . . . . . . . . . . . . . . . . . . . . . . . . . . . . . . . [1092]
8749401                    . . . . . . . . . . . . . . . G . . . . . T . . . . . . . . . . . . . . . . . . . . . . . . . . . . . . . . . . . . . . . . . . . . . . . . . . . . . . [1092]
8756347                    . . . . . . . . . . . . . . . G . . T . . T . . . . . . . . . . . . . . . . . . . . . . . . . . . . . . . . . . . . . . . . . . . . . . . . . . . . . . [1092]
AB3A                       . . . . . . . . . . . . . . . G . . . . . T . . . . . . . . . . . . . . . . . . . . . . . . . . . . . . . . . . . . . . . . . . . . . . . . . . . . . . [1092]
AB74A                      . . . . . . . . . . . . . . . G . . T . . T . . . . . . . . . . . . . . . . . . . . . . . . . . . . . . . . . . . . . . . . . . . . . . . . . . . . . . [1092]
AL 20-1                    . . . . . . . . . C . . . . . . . . T . . T . . . . . . . . . . . . . . . . . . . . . . . . . . . . . . . . . . . . . . . . . . . . . . . . . . . . . . [1092]
AO2A                       . . . . . . . . . . . . . . . G . . T . . T . . . . . . . . . . . . . . . . . . . . . . . . . . . . . . . . . . . . . . . . . . . . . . . . . . . . . . [1092]
BUF3                       . . . . . . . . . . . . . . . G . . . . . T . . . . . . . . . . . . . . . . . . . . . . . . . . . . . . . . . . . . . . . . . . . . . . . . . . . . . . [1092]
CV-152                     . . . . . . . . . . . . . . . . . . . . . . . . . . . . . . . . . . . . . . . . . . . . . . . . . . . . . . . . . . . . . . . . . . . . . . . . . . . . [1092]
CV-2101                    . . . . . . . . . . . . . . . . . . . . . . . . . . . . . . . . . . . . . . . . . . . . . . . . . . . . . . . . . . . . . . . . . . . . . . . . . . . . [1092]
EMU-3                      . . . . . . . . . . . . . . . . . . . . . . . . . . . . . . . . . . . . . . . . . . . . . . . . . . . . . . . . . . . . . . . . . . . . . . . . . . . . [1092]
F196                       . . . . . . . . . . . . . . . G . . . . . T . . . . . . . . . . . . . . . . . . . . . . . . . . . . . . . . . . . . . . . . . . . . . . . . . . . . . . [1092]
FE 7                       . . . . . . . . . C . . . . . G . . . . . . . . . . . . . . . . . . . . . . . . . . . . . . . . . . . . . . . . . . . . . . . . . . . . . . . . . . . . [1092]
FEBU4                      . . . . . . . . . . . . . . . G . . . . . T . . . . . . . . . . . . . . . . . . . . . . . . . . . . . . . . . . . . . . . . . . . . . . . . . . . . . . [1092]
HHS 118A                   . . . . . . . . . . . . . . . G . . . . . T . . . . . . . . . . . . . . . . . . . . . . . . . . . . . . . . . . . . . . . . . . . . . . . . . . . . . . [1092]
HHS 133                    . . . . . . . . . . . . . . . G . . . . . T . . . . . . . . . . . . . . . . . . . . . . . . . . . . . . . . . . . . . . . . . . . . . . . . . . . . . . [1092]
HHS 188A                   . . . . . . . . . . . . . . . G . . . . . T . . . . . . . . . . . . . . . . . . . . . . . . . . . . . . . . . . . . . . . . . . . . . . . . . . . . . . [1092]
HHS 191A                   . . . . . . . . . . . . . . . G . . T . . T . . . . . . . . . . . . . . . . . . . . . . . . . . . . . . . . . . . . . . . . . . . . . . . . . . . . . . [1092]
HHS 205A                   . . . . . . . . . . . . . . . G . . . . . T . . . . . . . . . . . . . . . . . . . . . . . . . . . . . . . . . . . . . . . . . . . . . . . . . . . . . . [1092]
LMG 10829                  . . . . . . . . . . . . . . . G . . T . . T . . . . . . . . . . . . . . . . . . . . . . . . . . . . . . . . . . . . . . . . . . . . . . . . . . . . . . [1092]
LMG 9863                   . . . . . . . . . C . . . . . G . . . . . . . . . . . . . . . . . . . . . . . . . . . . . . . . . . . . . . . . . . . . . . . . . . . . . . . . . . . . [1092]
MC 2-2                     . . . . . . . . . . . . . . . G . . . . . T . . . . . . . . . . . . . . . . . . . . . . . . . . . . . . . . . . . . . . . . . . . . . . . . . . . . . . [1092]
ME 15-4                    . . . . . . . . . C . . . . . G . . . . . . . . . . . . . . . . . . . . . . . . . . . . . . . . . . . . . . . . . . . . . . . . . . . . . . . . . . . . [1092]
MICV 42-1                  . . . . . . . . . . . . . . . G . . . . . T . . . . . . . . . . . . . . . . . . . . . . . . . . . . . . . . . . . . . . . . . . . . . . . . . . . . . . [1092]
NAV 12-2                   . . . . . . . . . . . . . . . G . . . . . T . . . . . . . . . . . . . . . . . . . . . . . . . . . . . . . . . . . . . . . . . . . . . . . . . . T . . . . . [1092]
NAV 15-1                   . . . . . . . . . C . . . . . . . . . . . . . . . . . . . . . . . . . . . . . . . . . . . . . . . . . . . . . . . . . . . . . . . . . . . . . . . . . . [1092]
NB14A                      . . . . . . . . . . . . . . . G . . T . . T . . . . . . . . . . . . . . . . . . . . . . . . . . . . . . . . . . . . . . . . . . . . . . . . . . . . . . [1092]
RW15-1                     . . . . . . . . . C . . . . . . . . . . . . . . . . . . . . . . . . . . . . . . . . . . . . . . . . . . . . . . . . . . . . . . . . . . . . . . . . . . [1092]
RW17-4                     . . . . . . . . . . . . . . . . . . . . . . . . . . . . . . . . . . . . . . . . . . . . . . . . . . . . . . . . . . . . . . . . . . . . . . . . . . . . [1092]
RW25-5                     . . . . . . . . . . . . . . . G . . . . . T . . . . . . . . . . . . . . . . . . . . . . . . . . . . . . . . . . . . . . . . . . . . . . . . . . . . . . [1092]
RW33-8                     . . . . . . . . . . . . . . . G . . . . . T . . . . . . . . . . . . . . . . . . . . . . . . . . . . . . . . . . . . . . . . . . . . . . . . . . . . . . [1092]
RW45-3                     . . . . . . . . . C . . . . . . . . . . . . . . . . . . . . . . . . . . . . . . . . . . . . . . . . . . . . . . . . . . . . . . . . . . . . . . . . . . [1092]
UF1T                       . . . . . . . . . . . . . . . G . . . . . T . . . . . . . . . . . . . . . . . . . . . . . . . . . . . . . . . . . . . . . . . . . . . . . . . . . . . . [1092]
UF2T                       . . . . . . . . . . . . . . . G . . . . . T . . . . . . . . . . . . . . . . . . . . . . . . . . . . . . . . . . . . . . . . . . . . . . . . . . . . . . [1092]
UPER3                      . . . . . . . . . . . . . . . G . . . . . T . . . . . . . . . . . . . . . . . . . . . . . . . . . . . . . . . . . . . . . . . . . . . . . . . . . . . . [1092]
A. trophiarum LMG25534T    T . . . . . T . . C . . . . . . . . T . . . . . . . . . . . . . . . . . . . . . . . . . . . . . . . . . . . . . . . . . . . . . . . . . . . . . . . . . [1092]
```

```
LMG24291T               TAAACCAACAGCTCCAATAGTTGGAACTGGTTTAGAAAAAACAGTTGCAAGAGATGCTTGGGAGGCTATAAAAGCTTC [1170]
LMG10241                ...............................................................A..A..T......AA [1170]
LMG10229                .............................................................................. [1170]
LMG9861                 ............................................................................AA [1170]
LMG9065                 .............................................................................. [1170]
LMG9871                 .............................................................................. [1170]
LMG 10210               .............................................................................. [1170]
LMG9865                 .............................................................................. [1170]
L397                    ...............................................................A..A..T......AA [1170]
L398                    ............A..................................................A..A..T......AA [1170]
L399                    ............A..................................................A..A..T......AA [1170]
L400                    ...............................................................A..A..T......AA [1170]
L401                    .............................................................................. [1170]
L406                    ...............................................................A..A..T......AA [1170]
14PHA                   ...............................................................A..A..T......AA [1170]
20PHF                   ...............................................................A..A..T......AA [1170]
284/1                   ...............................................................A..A..T......AA [1170]
8122333                 ...............................................................A..A..T......AA [1170]
8749401                 ...............................................................A..A..T......AA [1170]
8756347                 ...............................................................A..A..T......AA [1170]
AB3A                    ...........................A..AC.T........T....................A..A..T.......T [1170]
AB74A                   ...............................................................A..A..T......AA [1170]
AL 20-1                 .............................................................................. [1170]
AO2A                    ...............................................................A..A..T......AA [1170]
BUF3                    ...............................................................A..A..T......AA [1170]
CV-152                  .............................................................................. [1170]
CV-2101                 ...............................................................A.............. [1170]
EMU-3                   ...............................................................A.............. [1170]
F196                    ...............................................................A..A..T......AA [1170]
FE 7                    .............................................................................. [1170]
FEBU4                   .............................................................................. [1170]
HHS 118A                ...............................................................A..A..T......AA [1170]
HHS 133                 ...............................................................A..A..T......AA [1170]
HHS 188A                ...............................................................A..A..T......AA [1170]
HHS 191A                ...............................................................A..A..T......AA [1170]
HHS 205A                ...............................................................A..A..T......AA [1170]
LMG 10829               ...............................................................A..A..T......AA [1170]
LMG 9863                .............................................................................. [1170]
MC 2-2                  ...............................................................A..A..T......AA [1170]
ME 15-4                 .............................................................................. [1170]
MICV 42-1               ...........................A..AC.T........T....................A..A..T.......T [1170]
NAV 12-2                .............................................................................. [1170]
NAV 15-1                .............................................................................. [1170]
NB14A                   ...............................................................A..A..T......AA [1170]
RW15-1                  .............................................................................. [1170]
RW17-4                  .............................................................................. [1170]
RW25-5                  ...............................................................A..A..T......AA [1170]
RW33-8                  ...............................................................A..A..T......AA [1170]
RW45-3                  .............................................................................. [1170]
UF1T                    ...............................................................A..A..T......AA [1170]
UF2T                    ...............................................................A..A..T......AA [1170]
UPER3                   ...............................................................A..A..T......AA [1170]
A. trophiarum LMG25534T ............A.....T..............................C.T...........A...........CA. [1170]
```

LMG24291T	A A G A G G C G G A G T A G T A G A A A A A G C A G A T T C A A A A A A T A T C T A T A T T A G T G G T G A A G A T G A A A A A A T C A T C T T C A G T T G	[1248]
LMG10241	T . . . . . T . . . . . T . . T . . . . T . . . . T	[1248]
LMG10229	. . . . T . . . . T . . . . . C . . . . T	[1248]
LMG9861	T . . . . . T . . . . . T . . T . . . . T . . . . . C . . . . T	[1248]
LMG9065	. . . . A . . . . T	[1248]
LMG9871	. . . . A . . . . T	[1248]
LMG 10210	. . . . T . . . . G	[1248]
LMG9865	. . . . T . . . . G	[1248]
L397	T . . . . . T . . . . . T . . T . . . . T . . . . . C . . . . T	[1248]
L398	T . . . . . T . . . . . T . . T . . . . T . . . . . C . . . . T	[1248]
L399	T . . . . . T . . . . . T . . T . . . . T . . . . . C . . . . T	[1248]
L400	T . . . . . T . . . . . T . . T . . . . T . . . . . C . . . . T	[1248]
L401	. . . . . . T . . . . . T . . T . . . . T . . . . . C . . . . T	[1248]
L406	T . . . . . T . . . . . T . . T . . . . T . . . . . C . . . . T	[1248]
14PHA	T . . . . . T . . G . . T . . T . . . . T . . . . . C . . . . T	[1248]
20PHF	T . . . . . T . . . . . T . . T . . . . T . . . . . C . . . . T	[1248]
284/1	T . . . . . T . . . . . T . . T . . . . T . . . . . C . . . . C . . . . T	[1248]
8122333	T . . . . . T . . . . . T . . T . . . . T . . . . . C . . . . T	[1248]
8749401	T . . . . . T . . . . . T . . T . . . . T . . . . . C . . . . T	[1248]
8756347	T . . . . . T . . . . . T . . T . . . . T . . . . . C . . . . T	[1248]
AB3A	. . . . . . T . . . . . T . . T . . . . T . . C . . T	[1248]
AB74A	T . . . . . T . . . . . T . . T . . . . T . . . . . C . . . . C . . . . T	[1248]
AL 20-1	. . . . A . T . . . . T	[1248]
AO2A	T . . . . . T . . . . . T . . T . . . . T . . . . . C . . . . T	[1248]
BUF3	T . . . . . T . . . . . T . . T . . . . T . . . . . C . . . . T	[1248]
CV-152	. . . .	[1248]
CV-2101	. . . .	[1248]
EMU-3	. . . .	[1248]
F196	T . . . . . T . . . . . T . . T . . . . T . . . . . C . . . . T	[1248]
FE 7	. . . . A . T . . . . T	[1248]
FEBU4	. . . . . . T . . . . . T . . T . . . . C	[1248]
HHS 118A	T . . . . . T . . . . . T . . T . . . . T . . . . . C . . . . T	[1248]
HHS 133	T . . . . . T . . . . . T . . T . . . . T . . . . . C . . . . T	[1248]
HHS 188A	T . . . . . T . . . . . T . . T . . . . T . . . . . C . . . . T	[1248]
HHS 191A	T . . . . . T . . . . . T . . T . . . . T . . . . . C	[1248]
HHS 205A	T . . . . . T . . . . . T . . T . . . . T . . . . . C . . . . T	[1248]
LMG 10829	T . . . . . T . . . . . T . . T . . . . T . . . . . C . . . . T	[1248]
LMG 9863	. . . . A . . . . T	[1248]
MC 2-2	T . . . . . T . . . . . T . . T . . . . T . . . . . C . . . . C . . . . T	[1248]
ME 15-4	. . . . A . T . . . . T	[1248]
MICV 42-1	. . . . . . T . . . . . T . . T . . . . T . . C . . T	[1248]
NAV 12-2	. . . . . . T . . . . . T . . T . . . . T . . . . . C . . . . T	[1248]
NAV 15-1	. . . . T . . . . G	[1248]
NB14A	T . . . . . T . . . . . T . . T . . . . T . . . . . C . . . . T	[1248]
RW15-1	. . . . T . . . . G	[1248]
RW17-4	. . . . T . . . . G	[1248]
RW25-5	T . . . . . T . . . . . T . . T . . . . T . . . . . C	[1248]
RW33-8	T . . . . . T . . . . . T . . T . . . . T . . . . . C . . . . T	[1248]
RW45-3	. . . . T . . . . G	[1248]
UF1T	T . . . . . T . . . . . T . . T . . . . T . . . . . C . . . . T	[1248]
UF2T	T . . . . . T . . . . . T . . T . . . . T . . . . . C . . . . T	[1248]
UPER3	T . . . . . T . . . . . T . . T . . . . T . . . . . C . . . . T	[1248]
A. trophiarum LMG25534T	T . . . . . T . . . . T . . . . T	[1248]

LMG24291T	C T T C T G T T G T T A G A A C A T T A G A A G A T G C A G G A G C T A T G G A T T A T A C A A T T G T T G T A A A T G C A T C A G C A G C T G A T T C T G	[1326]
LMG10241	. . . . A . . . . . A . . . . . . C . T . . . . . A . . T . . . . T T . . . . A T	[1326]
LMG10229	. . . . A . . . . . G . . . . . . C . T . . . . . A . . T . . . . T T . . . . A T	[1326]
LMG9861	. . . . T . . . . T	[1326]
LMG9065	. . . . T . . . . T	[1326]
LMG9871	. . . . T . . . . T	[1326]
LMG 10210	. . . . T . . . . T . . G	[1326]
LMG9865	. . . . T . . . . T . . G	[1326]
L397	. . . . A . . . . . A . . . . . . C . T . . . . . A . . T . . . . T T . . . . A T	[1326]
L398	. . . . A . . . . . A . . . . . . C . T . . . . . A . . T . . . . T T . . . . A T	[1326]
L399	. . . . A . . . . . A . . . . . . C . T . . . . . A . . T . . . . T T . . . . A T	[1326]
L400	. . . . A . . . . . A . . . . . . C . T . . . . . A . . T . . . . T T . . . . A T	[1326]
L401	. . . . A . . . . . A . . . . . . C . T . . . . . A . . T . . . . T T . . . . A T	[1326]
L406	. . . . A . . . . . A . . . . . . C . T . . . . . A . . T . . . . T T . . . . A T	[1326]
14PHA	. . . . A . . . . . A . . . . . . C . T . . . . . A . . T . . . . T T . . . . A T	[1326]
20PHF	. . . . A . . . . . A . . . . . . C . T . . . . . A . . T . . . . T T . . . . A T	[1326]
284/1	. . . . A . . . . . A . . . . . . C . T . . . . . A . . T . . . . T T . . . . A T	[1326]
8122333	. . . . A . . . . . A . . . . . . C . T . . . . . A . . T . . . . T T . . . . A T	[1326]
8749401	. . . . A . . . . . A . . . . . . C . T . . . . . A . . T . . . . T T . . . . A T	[1326]
8756347	. . . . A . . . . . A . . . . . . C . T . . . . . A . . T . . . . T T . . . . A T	[1326]
AB3A	. . . . T . . . . C . . . . T . . . . . C . . A .	[1326]
AB74A	. . . . A . . . . . A . . . . . . C . T . . . . . A . . T . . . . T T . . . . A T	[1326]
AL 20-1	. . . . T . . . . T	[1326]
AO2A	. . . . A . . . . . A . . . . . . C . T . . . . . A . . T . . . . T T . . . . A T	[1326]
BUF3	. . . . A . . . . . A . . . . . . C . T . . . . . A . . T . . . . T T . . . . A T	[1326]
CV-152	. . . . G	[1326]
CV-2101	. . . . G	[1326]
EMU-3	. . . . G	[1326]
F196	. . . . A . . . . . A . . . . . . C . T . . . . . A . . T . . . . T T . . . . A T	[1326]
FE 7	. . . . T . . . . T	[1326]
FEBU4	. . . . T . . . . T . . G	[1326]
HHS 118A	. . . . A . . . . . A . . . . . . C . T . . . . . A . . T . . . . T T . . . . A T	[1326]
HHS 133	. . . . A . . . . . A . . . . . . C . T . . . . . A . . T . . . . T . . . . T . . G	[1326]
HHS 188A	. . . . A . . . . . A . . . . . . C . T . . . . . A . . T . . . . T T . . . . A T	[1326]
HHS 191A	. . . . A . . . . . A . . . . . . C . T . . . . . A . . . . T T . . . . A T	[1326]
HHS 205A	. . . . A . . . . . A . . . . . . C . T . . . . . A . . T . . . . T . . . . T T . . . . A T	[1326]
LMG 10829	. . . . A . . . . . A . . . . . . C . T . . . . . A . . T . . . . T T . . . . A T	[1326]
LMG 9863	. . . . T	[1326]
MC 2-2	. . . . A . . . . . A . . . . . . C . T . . . . . A . . T . . . . T T . . . . A T	[1326]
ME 15-4	. . . . T . . . . T	[1326]
MICV 42-1	. . . . T . . . . C . . . . T . . . . . C . . A .	[1326]
NAV 12-2	. . . . A . . . . . G . . . . . . C . T . . . . . A . . T . . . . T T . . . . A T	[1326]
NAV 15-1	. . . . G . . . . T . . G	[1326]
NB14A	. . . . A . . . . . A . . . . . . C . T . . . . . A . . T . . . . T T . . . . A T	[1326]
RW15-1	. . . . T . . . . T . . G	[1326]
RW17-4	. . . . T . . . . T . . G	[1326]
RW25-5	. . . . T . . . . C . . . . T	[1326]
RW33-8	. . . . A . . . . . A . . . . . . C . T . . . . . A . . T . . . . T T . . . . A T	[1326]
RW45-3	. . . . T . . . . T . . G	[1326]
UF1T	. . . . A . . . . . A . . . . . . C . T . . . . . A . . T . . . . T T . . . . A T	[1326]
UF2T	. . . . A . . . . . A . . . . . . C . T . . . . . A . . T . . . . T T . . . . A T	[1326]
UPER3	. . . . A . . . . . A . . . . . . C . T . . . . . A . . T . . . . T . . . . T T . . . . A T	[1326]
A. trophiarum LMG25534T	. . . . A . . . . . A . . . . . . C . T . . . . . A . . . . A .	[1326]

LMG24291T	C A A C A C T T C A A T T C T T A G C A C C A T A T A C A G G T G T T A C A A T T G G T G A G T T T T T C A G A G A T A A T G G A A A A C A T G C A C T T A	[1404]
LMG10241	. . . . . T . A . . . . . . C . T . . . . T . . G . . . . . A . . . . T . . . . A .	[1404]
LMG10229	. . . . . T . A . . . . . . C . T . . . . T . . G . . . . T . . . . A .	[1404]
LMG9861	. . . . G . . . . T C . . . . A . . C . . . . T	[1404]
LMG9065	. . . . T	[1404]
LMG9871	. . . . T	[1404]
LMG 10210	. . . . G . . . . T C . . . . A . . C . . . . C	[1404]
LMG9865	. . . . G . . . . T C . . . . A . . C . . . . C	[1404]
L397	. . . . . T . A . . . . . . C . T . . . . T . . G . . . . . A . . . . T . . . . A .	[1404]
L398	. . . . . T . A . . . . . . C . T . . . . T . . G . . . . . A . . . . T . . G . . . . A .	[1404]
L399	. . . . . T . A . . . . . T C . T . . . . T . . G . . . . . A . . . . T . . . . A .	[1404]
L400	. . . . . T . A . . . . . . C . T . . . . T . . G . . . . . A . . . . T . . . . A .	[1404]
L401	. . . . . T . A . . . . . . C . T . . . . T . . G . . . . . A . . . . T . . G . . . . A .	[1404]
L406	. . . . . T . A . . . . . . C . T . . . . T . . G . . . . . A . . . . T . . . . A .	[1404]
14PHA	. . . . . T . A . . . . . . C . T . . . . T . . G . . . . . A . . . . T . . . . A .	[1404]
20PHF	. . . . . T . A . . . . . . C . T . . . . T . . G . . . . . A . . . . T . . . . A .	[1404]
284/1	. . . . . T . A . . . . . . C . T . . . . T . . G . . . . . A . . . . T . . . . T . A .	[1404]
8122333	. . . . . T . A . . . . . . C . T . . . . T . . G . . . . . A . . . . T . . . . T . A .	[1404]
8749401	. . . . . T . A . . . . . . C . T . . . . T . . G . . . . . A . . . . T . . . . A .	[1404]
8756347	. . . . . T . A . . . . . . C . T . . . . T . . G . . . . . A . . . . T . . . . T . A .	[1404]
AB3A	. . . . . T . A . . . . . T . . G . . . . A . . C . . T . . . . C . . . . . A .	[1404]
AB74A	. . . . . T . A . . . . . . C . T . . . . T . . G . . . . . A . . . . T . . . . A .	[1404]
AL 20-1	. . . . T . . . . C	[1404]
AO2A	. . . . . T . A . . . . . . C . T . . . . T . . G . . . . . A . . . . T . . . . T . A .	[1404]
BUF3	. . . . . T . A . . . . . . C . T . . . . T . . G . . . . . A . . . . T . . . . A .	[1404]
CV-152	. . . . C	[1404]
CV-2101	. . . . C	[1404]
EMU-3	. . . . C	[1404]
F196	. . . . . T . A . . . . . . C . T . . . . T . . G . . . . . A . . . . T . . . . T . A .	[1404]
FE 7	. . . . T . . . . C	[1404]
FEBU4	. . . . G . . . . T C . . . . A . . C . . . . C	[1404]
HHS 118A	. . . . . T . A . . . . . . C . T . . . . T . . G . . . . . A . . . . T . . . . A .	[1404]
HHS 133	. . . . G . . . . T C . . . . T . . G . . . . . A . . C . . T . . . . T . . . . A .	[1404]
HHS 188A	. . . . . T . A . . . . . . C . T . . . . T . . G . . . . . A . . . . T . . . . A .	[1404]
HHS 191A	. . . . . T . A . . . . . . C . T . . . . T . . G . . . . . A . . . . T . . . . A .	[1404]
HHS 205A	. . . . . T . A . . . . . . C . T . . . . A . . C . . . . C	[1404]
LMG 10829	. . . . . T . A . . . . . . C . T . . . . T . . G . . . . . A . . . . T . . . . A .	[1404]
LMG 9863	. . . . T	[1404]
MC 2-2	. . . . . T . A . . . . . . C . T . . . . T . . G . . . . . A . . . . T . . G . . . . A .	[1404]
ME 15-4	. . . . T . . . . C	[1404]
MICV 42-1	. . . . . T . A . . . . . T . . G . . . . A . . C . . T . . . . C . . . . . A .	[1404]
NAV 12-2	. . . . . T . A . . . . . . C . T . . . . T . . G . . . . T . . . . A .	[1404]
NAV 15-1	. . . . G . . . . T C . . . . A . . C . . . . C	[1404]
NB14A	. . . . . T . A . . . . . . C . T . . . . T . . G . . . . . A . . . . T . . . . T . A .	[1404]
RW15-1	. . . . G . . . . T C . . . . A . . C . . . . C	[1404]
RW17-4	. . . . G . . . . T C . . . . A . . C . . . . C	[1404]
RW25-5	. . . . G . . . . T C . . . . A . . C . . . . C	[1404]
RW33-8	. . . . . T . A . . . . . . C . T . . . . T . . G . . . . . A . . . . . T . . . . T . . . . A .	[1404]
RW45-3	. . . . G . . . . T C . . . . A . . C . . . . C	[1404]
UF1T	. . . . . T . A . . . . . . C . T . . . . T . . G . . . . . A . . . . T . . . . A .	[1404]
UF2T	. . . . . T . A . . . . . . C . T . . . . T . . G . . . . . A . . . . T . . . . A .	[1404]
UPER3	. . . . . T . A . . . . . . C . T . . . . T . . G . . . . . A . . . . T . . . . A .	[1404]
A. trophiarum LMG25534T	. . . . . T . A . . . . . T . . . . C . . T . . . . T	[1404]

LMG24291T	T T A T T T A T G A T G A T T T A T C A A A A C A T G C A G T T G C A T A T A G A G A G A T G T C A T T A A T T T T A A G A A G A C C T C C A G G A A G A G	[1482]
LMG10241	. . . .	[1482]
LMG10229	. . . .	[1482]
LMG9861	. . . .	[1482]
LMG9065	. . . . A . . . . G	[1482]
LMG9871	. . . . A . . . . G	[1482]
LMG 10210	. . . .	[1482]
LMG9865	. . . .	[1482]
L397	. . . .	[1482]
L398	. . . .	[1482]
L399	. . . .	[1482]
L400	. . . .	[1482]
L401	. . . .	[1482]
L406	. . . .	[1482]
14PHA	. . . .	[1482]
20PHF	. . . .	[1482]
284/1	. . . .	[1482]
8122333	. . . .	[1482]
8749401	. . . .	[1482]
8756347	. . . .	[1482]
AB3A	. . . .	[1482]
AB74A	. . . .	[1482]
AL 20-1	. . . . A . . . . G	[1482]
AO2A	. . . .	[1482]
BUF3	. . . .	[1482]
CV-152	. . . .	[1482]
CV-2101	. . . .	[1482]
EMU-3	. . . .	[1482]
F196	. . . .	[1482]
FE 7	. . . . A . . . . G	[1482]
FEBU4	. . . .	[1482]
HHS 118A	. . . .	[1482]
HHS 133	. . . .	[1482]
HHS 188A	. . . .	[1482]
HHS 191A	. . . .	[1482]
HHS 205A	. . . .	[1482]
LMG 10829	. . . .	[1482]
LMG 9863	. . . . A	[1482]
MC 2-2	. . . .	[1482]
ME 15-4	. . . . A . . . . G	[1482]
MICV 42-1	. . . .	[1482]
NAV 12-2	. . . .	[1482]
NAV 15-1	. . . .	[1482]
NB14A	. . . .	[1482]
RW15-1	. . . .	[1482]
RW17-4	. . . .	[1482]
RW25-5	. . . .	[1482]
RW33-8	. . . .	[1482]
RW45-3	. . . .	[1482]
UF1T	. . . .	[1482]
UF2T	. . . .	[1482]
UPER3	. . . .	[1482]
A. trophiarum LMG25534T	. . . .	[1482]

LMG24291T	A A G C A T A T C C A G G A G A T G T A T T C T A T T T A C A C T C A A G A T T A C T T G A A A G A G C A G C T A A A A T G A G T G A T G A A A A A G G T G	[1560]
LMG10241	. . . . C . . . . C . . . . C C . . . . G . . . . . T . . A	[1560]
LMG10229	. . . . C . . . . C . . . . C C . . . . G . . . . . T	[1560]
LMG9861	. . . . C . . . . C . . . . C C . . . . G . . . . . T . . A	[1560]
LMG9065	. . . . C C . . . . T . . A	[1560]
LMG9871	. . . . C C . . . . G . . . . . T . . A	[1560]
LMG 10210	. . . . C C . . . . G . . . . . T	[1560]
LMG9865	. . . . C C . . . . G . . . . . T	[1560]
L397	. . . . C . . . . C . . . . . T . . C C . . . . G . . . . . T . . A	[1560]
L398	. . . . C . . . . C . . . . C C . . . . G . . . . . T	[1560]
L399	. . . . C . . . . C . . . . C C . . . . G . . . . . T . . A	[1560]
L400	. . . . C . . . . C . . . . C C . . . . G . . . . . T . . A	[1560]
L401	. . . . C . . . . C . . . . C C . . . . G . . . . . T	[1560]
L406	. . . . C . . . . C . . . . C C . . . . G . . . . . T . . A	[1560]
14PHA	. . . . C . . . . C C . . . . G . . . . . T . . A	[1560]
20PHF	. . . . C . . . . C . . . . C C . . . . G . . . . . T . . A	[1560]
284/1	. . . . C . . . . C . . . . C C . . . . G . . . . . T . . A	[1560]
8122333	. . . . C . . . . C . . . . C C . . . . G . . . . . T . . A	[1560]
8749401	. . . . C . . . . C . . . . C C . . . . G . . . . . T . . A	[1560]
8756347	. . . . C . . . . C . . . . C C . . . . G . . . . . T . . A	[1560]
AB3A	. . . . T . . . . C . G . . . . T . . A	[1560]
AB74A	. . . . C . . . . C . . . . C C . . . . G . . . . . T . . A	[1560]
AL 20-1	. . . . C . . . . C C . . . . G . . . . . T . . A	[1560]
AO2A	. . . . C . . . . C . . . . C C . . . . G . . . . . T . . A	[1560]
BUF3	. . . . C . . . . C . . . . C C . . . . G . . . . . T . . A	[1560]
CV-152	. . . . C	[1560]
CV-2101	. . . . C	[1560]
EMU-3	. . . . C	[1560]
F196	. . . . C . . . . C . . . . C C . . . . G . . . . . T . . A	[1560]
FE 7	. . . . C . . . . C C . . . . G . . . . . T . . A	[1560]
FEBU4	. . . . C C . . . . G . . . . . T	[1560]
HHS 118A	. . . . C . . . . C . . . . C C . . . . G . . . . . T . . A	[1560]
HHS 133	. . . . C . . . . C . . . . C C . . . . G . . . . . T	[1560]
HHS 188A	. . . . C . . . . C . . . . C C . . . . G . . . . . T . . A	[1560]
HHS 191A	. . . . C . . . . C . . . . C C . . . . G . . . . . T . . A	[1560]
HHS 205A	. . . . C . . . . C . . . . C C . . . . G . . . . . T	[1560]
LMG 10829	. . . . C . . . . C . . . . C C . . . . G . . . . . T . . A	[1560]
LMG 9863	. . . . C C . . . . T . . A	[1560]
MC 2-2	. . . . C . . . . C . . . . C C . . . . G . . . . . T . . A	[1560]
ME 15-4	. . . . C . . . . C C . . . . G . . . . . T . . A	[1560]
MICV 42-1	. . . . T . . . . C . G . . . . T . . A	[1560]
NAV 12-2	. . . . C . . . . C . . . . C C . . . . G . . . . . T . . A	[1560]
NAV 15-1	. . . . C C . . . . G C . . . . T	[1560]
NB14A	. . . . C . . . . C . . . . C C . . . . G . . . . . T . . A	[1560]
RW15-1	. . . . C . . . . G . . . . . T	[1560]
RW17-4	. . . . C C . . . . G . . . . . T	[1560]
RW25-5	. . . . T . . . . C . G . . . . T . . A	[1560]
RW33-8	. . . . C . . . . C . . . . C C . . . . G . . . . . T . . A	[1560]
RW45-3	. . . . C C . . . . G . . . . . T	[1560]
UF1T	. . . . C . . . . C . . . . C C . . . . G . . . . . T . . A	[1560]
UF2T	. . . . C . . . . C . . . . C C . . . . G . . . . . T	[1560]
UPER3	. . . . C . . . . C . . . . C C . . . . G . . . . . T	[1560]
A. trophiarum LMG25534T	. . . . C . . . . C . . . . . T . . . C . . . . T	[1560]

LMG24291T	C T G G T T C A A T G A C T G C G C T A C C A A T T A T T G A A A C T C A A G C T G G A G A T G T T G C T G C A T A T A T T C C A A C A A A C G T A A T T T	[1638]
LMG10241	. . . . T . . . . T . . . . C	[1638]
LMG10229	. . . . T . . . . T . . . . C . . . . C	[1638]
LMG9861	. . . . T . . . . T . . . . C	[1638]
LMG9065	. . . . C	[1638]
LMG9871	. . . . C	[1638]
LMG 10210	. . . . C	[1638]
LMG9865	. . . . C	[1638]
L397	. . . . T . . . . T . . . . C	[1638]
L398	. . . . T . . . . T . . . . C	[1638]
L399	. . . . T . . . . T . . . . C	[1638]
L400	. . . . T . . . . T . . . . T . . . . C	[1638]
L401	. . . . T . . . . T . . . . C	[1638]
L406	. . . . T . . . . T . . . . C	[1638]
14PHA	. . . . T . . . . A T . . . . C	[1638]
20PHF	. . . . T . . . . T . . . . C	[1638]
284/1	. . . . T . . . . T . . . . C	[1638]
8122333	. . . . T . . . . T . . . . C	[1638]
8749401	. . . . T . . . . T . . . . C	[1638]
8756347	. . . . T . . . . T . . . . C	[1638]
AB3A	. . . . T . . . . T . . . . C	[1638]
AB74A	. . . . T . . . . T . . . . C	[1638]
AL 20-1	. . . . C	[1638]
AO2A	. . . . T . . . . T . . . . C	[1638]
BUF3	. . . . T . . . . A T . . . . C	[1638]
CV-152	. . . .	[1638]
CV-2101	. . . . G . . T	[1638]
EMU-3	. . . . G . . T	[1638]
F196	. . . . T . . . . T . . . . T . . . . C	[1638]
FE 7	. . . . C	[1638]
FEBU4	. . . . C	[1638]
HHS 118A	. . . . T . . . . C T . . . . C	[1638]
HHS 133	. . . . T . . . . C	[1638]
HHS 188A	. . . . T . . . . T . . . . C	[1638]
HHS 191A	. . . . T . . . . T . . . . T . . . . C	[1638]
HHS 205A	. . . . T . . . . T . . . . C	[1638]
LMG 10829	. . . . T . . . . T . . . . C	[1638]
LMG 9863	. . . . C	[1638]
MC 2-2	. . . . T . . . . T . . . . T . . . . C . . . . T	[1638]
ME 15-4	. . . . C	[1638]
MICV 42-1	. . . . T . . . . T . . . . C	[1638]
NAV 12-2	. . . . T . . . . T . . . . C . . . . T	[1638]
NAV 15-1	. . . . C	[1638]
NB14A	. . . . T . . . . T . . . . C	[1638]
RW15-1	. . . . C	[1638]
RW17-4	. . . . C	[1638]
RW25-5	. . . . T . . . . T . . . . T . . . . C	[1638]
RW33-8	. . . . T . . . . T . . . . T . . . . C	[1638]
RW45-3	. . . . C	[1638]
UF1T	. . . . T . . . . T . . . . C . . . . T	[1638]
UF2T	. . . . T . . . . T . . . . C	[1638]
UPER3	. . . . T . . . . T . . . . C	[1638]
A. trophiarum LMG25534T	. . . . A . . . . A . . . . . T . . . . C	[1638]

LMG24291T	C A A T T A C A G A T G G A C A A A T T T T C T T A G A A A C T A A C C T A T T T A A C T C A G G T A T T A G A C C T G C A A T T A A T G T T G G T T T A T	[1716]
LMG10241	. T . . . . . T . . . . A . . . . A . . . . C	[1716]
LMG10229	. . . . T . . . . A . . . . A . . . . C	[1716]
LMG9861	. T . . . . . T . . . . A . . . . A . . . . C	[1716]
LMG9065	. . . . T	[1716]
LMG9871	. . . . T	[1716]
LMG 10210	. G . . . . A . . . . T . . . . . C . . A . . A C . T .	[1716]
LMG9865	. G . . . . A . . . . T . . . . . C . . A . . A C . T .	[1716]
L397	. T . . . . . T . . . . A . . . . A . . . . C	[1716]
L398	. . . . T . . . . T	[1716]
L399	. T . . . . . T . . . . A . . . . A . . . . C	[1716]
L400	. T . . . . . T . . . . A . . . . A . . . . C	[1716]
L401	. . . . T . . . . T	[1716]
L406	. T . . . . . T . . . . A . . . . A . . . . C	[1716]
14PHA	. T . . . . . T . . . . A . . . . A . . . . C	[1716]
20PHF	. T . . . . . T . . . . A . . . . A . . . . C	[1716]
284/1	. T . . . . . T . . . . A . . . . A . . . . C	[1716]
8122333	. T . . . . . T . . . . A . . . . A . . . . C	[1716]
8749401	. T . . . . . T . . . . A . . . . A . . . . C	[1716]
8756347	. T . . . . . T . . . . A . . . . A . . . . C	[1716]
AB3A	. T . . . . . T . . . . T	[1716]
AB74A	. T . . . . . T . . . . A . . . . A . . . . C	[1716]
AL 20-1	. . . . T	[1716]
AO2A	. T . . . . . T . . . . A . . . . A . . . . C	[1716]
BUF3	. T . . . . . T . . . . A . . . . A . . . . C	[1716]
CV-152	. . . . T	[1716]
CV-2101	. . . .	[1716]
EMU-3	. . . .	[1716]
F196	. T . . . . . T . . . . A . . . . A . . . . C	[1716]
FE 7	. . . . T	[1716]
FEBU4	. G . . . . A . . . . T . . . . . C . . A . . A C . T .	[1716]
HHS 118A	. T . . . . . T . . . . A . . . . A . . . . C	[1716]
HHS 133	. T . . . . . T . . . . A . . . . A . . . . C	[1716]
HHS 188A	. T . . . . . T . . . . A . . . . A . . . . C	[1716]
HHS 191A	. T . . . . . T . . . . A . . . . A . . . . C	[1716]
HHS 205A	. T . . . . A . . . . A . . . . C	[1716]
LMG 10829	. T . . . . . T . . . . A . . . . A . . . . C	[1716]
LMG 9863	. . . . T	[1716]
MC 2-2	. T . . . . . T . . . . A . . . . A . . . . C	[1716]
ME 15-4	. . . . T	[1716]
MICV 42-1	. T . . . . . T . . . . T	[1716]
NAV 12-2	. T . . . . . T . . . . A . . . . A . . . . C	[1716]
NAV 15-1	. G . . . . A . . . . A . . . . C	[1716]
NB14A	. T . . . . . T . . . . A . . . . A . . . . C	[1716]
RW15-1	. G . . . . A . . . . C	[1716]
RW17-4	. G . . . . A . . . . T . . . . . C . . A . . A C . T .	[1716]
RW25-5	. . . . T . . . . T	[1716]
RW33-8	. T . . . . . T . . . . A . . . . A . . . . C	[1716]
RW45-3	. G . . . . A . . . . T . . . . . C . . A . . A C . T .	[1716]
UF1T	. T . . . . . T . . . . A . . . . A . . . . C	[1716]
UF2T	. T . . . . . T . . . . A . . . . A . . . . C	[1716]
UPER3	. T . . . . . T . . . . A . . . . A . . . . C	[1716]
A. trophiarum LMG25534T	. . . . A . . . C . . . . A . . . . A . . . . T	[1716]

LMG24291T	C A G T A T C A A G A G T T G G T G G A G C T G C A C A A A T T A A A G C T A C A A A A C A A G T T G C T G G T A C A C T T A A A C T T T C A C T T G C A C	[1794]
LMG10241	. . . . T . . . . A . . . . T . . . . T . A . . . . . A . . T	[1794]
LMG10229	. . . . T . . . . T . . . . G	[1794]
LMG9861	. . . . T . . . . A . . . . T . . . . T . A . . . . . A . . T	[1794]
LMG9065	. . . .	[1794]
LMG9871	. . . .	[1794]
LMG 10210	. T . . . . T	[1794]
LMG9865	. T . . . . T	[1794]
L397	. . . . T . . . . T . A . . . . . A . . T	[1794]
L398	. . . . T . . . . T . . . . C . . . . G	[1794]
L399	. . . . T . . . . A . . . . T . . . . T . A . . . . . A . . T	[1794]
L400	. . . . T . . . . A . . . . T . . . . T . A . . . . . A . . T	[1794]
L401	. . . . T . . . . T . . . . G	[1794]
L406	. . . . T . . . . T . A . . . . . A . . T	[1794]
14PHA	. . . . T . . . . A . . . . T . . . . T . A . . . . . A . . T	[1794]
20PHF	. . . . T . . . . T . . . . T . A . . . . . A . . T	[1794]
284/1	. . . . T . . . . T . . . . T . A . . . . . A . . T	[1794]
8122333	. . . . T . . . . A . . . . T . . . . T . A . . . . . A . . T	[1794]
8749401	. . . . T . . . . G	[1794]
8756347	. . . . T . . . . A . . . . T . . . . G . . . . T . A . . . . . A . . T	[1794]
AB3A	. . . . T . . . . T	[1794]
AB74A	. . . . T . . . . A . . . . T . . . . T . A . . . . . A . . T	[1794]
AL 20-1	. . . .	[1794]
AO2A	. . . . T . . . . A . . . . T . . . . T . A . . . . . A . . T	[1794]
BUF3	. . . . T . . . . A . . . . T . . . . T . A . . . . . A . . T	[1794]
CV-152	. . . .	[1794]
CV-2101	. . . .	[1794]
EMU-3	. . . .	[1794]
F196	. . . . T . . . . T . A . . . . . A . . T	[1794]
FE 7	. . . .	[1794]
FEBU4	. T . . . . T	[1794]
HHS 118A	. . . . T . . . . A . . . . T . . . . T . A . . . . . A . . T	[1794]
HHS 133	. . . . T . . . . A . . . . T . . . . T . A . . . . . A . . T	[1794]
HHS 188A	. . . . T . . . . G	[1794]
HHS 191A	. . . . T . . . . A . . . . T . . . . T . A . . . . . A . . T . . . . . G .	[1794]
HHS 205A	. . . . T . . . . T	[1794]
LMG 10829	. . . . T . . . . G	[1794]
LMG 9863	. . . .	[1794]
MC 2-2	. . . . T . . . . T . A . . . . . A . . T	[1794]
ME 15-4	. . . .	[1794]
MICV 42-1	. . . . T . . . . T	[1794]
NAV 12-2	. . . . T . . . . A . . . . T . . . . T . A . . . . . A . . T	[1794]
NAV 15-1	. . . . T . . . . A . . . . T . A . . . . . A . . T	[1794]
NB14A	. . . . T . . . . A . . . . T . . . . T . A . . . . . A . . T	[1794]
RW15-1	. . . . T . . . . A	[1794]
RW17-4	. T . . . . T	[1794]
RW25-5	. . . . T . . . . T	[1794]
RW33-8	. . . . T . . . . A . . . . T . . . . T . A . . . . . A . . T	[1794]
RW45-3	. T . . . . T	[1794]
UF1T	. . . . T . . . . T . A . . . . . A . . T	[1794]
UF2T	. . . . T . . . . T . . . . T . A . . . . . A . . T	[1794]
UPER3	. . . . T . . . . A . . . . T . A . . . . . A . . T	[1794]
A. trophiarum LMG25534T	. . . . T . . . . . G . . . . T . . . . T . A . . . . T . . . . . T .	[1794]

LMG24291T	AATATAGAGAGCTTGAGGCGTTTGCACAATTCGCATCAGATCTAGATGAAGCAACAAGAAGCAACTATTTTAGCTCAC	[1872]
LMG10241	..........A...AG...........	[1872]
LMG10229	..........A.....A...................................T.............AG...........	[1872]
LMG9861	..........A.....A...	[1872]
LMG9065	................A...	[1872]
LMG9871	................A...	[1872]
LMG 10210	....C..............G......................	[1872]
LMG9865	....C..............G......................	[1872]
L397	..........A.....A...G...........	[1872]
L398	..........A..C..A...................................T.............G...........	[1872]
L399	..........A.....A...................................T.............G...........	[1872]
L400	................A...G...........	[1872]
L401	..........A..C..A...................................T.............G...........	[1872]
L406	..........A...AG...........	[1872]
14PHA	..........A.....A...G...........	[1872]
20PHF	..........A.....A...G...........	[1872]
284/1	..........A.....A..AG...........	[1872]
8122333	..........A....................T..................................G...........	[1872]
8749401	.......G..A..AG...........	[1872]
8756347	.......G..A..AG...........	[1872]
AB3A	..........A.....A...................................T............AG...........	[1872]
AB74A	..........A...G...........	[1872]
AL 20-1	................A...	[1872]
AO2A	..........A....................T.................................AG...........	[1872]
BUF3	..........A.....A..AG...........	[1872]
CV-152	....	[1872]
CV-2101	....	[1872]
EMU-3	....	[1872]
F196	..........A..AG...........	[1872]
FE 7	................A...	[1872]
FEBU4	....C..............G.........AG...........	[1872]
HHS 118A	..........A.....A...G...........	[1872]
HHS 133	..........A.....A...G...........	[1872]
HHS 188A	.......G..A..AG...........	[1872]
HHS 191A	..........A...G...........	[1872]
HHS 205A	....C.........................G...........	[1872]
LMG 10829	.......G..A...G...........	[1872]
LMG 9863	................A...C...	[1872]
MC 2-2	..........A...G...........	[1872]
ME 15-4	................A........................T....................................	[1872]
MICV 42-1	..........A.....A...................................T.............G...........	[1872]
NAV 12-2	..........A.....A...................................T.............G...........	[1872]
NAV 15-1	....C..............G......................	[1872]
NB14A	..........A....................T.................................AG...........	[1872]
RW15-1	....C..............G......................	[1872]
RW17-4	....C..............G......................	[1872]
RW25-5	..........A.....A...................................T.........................	[1872]
RW33-8	..........A..AG...........	[1872]
RW45-3	....C..............G......................	[1872]
UF1T	..........A.....A...G...........	[1872]
UF2T	..........A..AG...........	[1872]
UPER3	..........A.....A..AG...........	[1872]
A. trophiarum LMG25534T	................A..C........G..T.........T.......................AG...........	[1872]

LMG24291T	T C A A T T T T C A A A G A A G G T C T T A G A A A C G T A A C A G C A G G A G C A A A T C C A A T A T C T T T A A A A A G A G G T A T G G A T A A A G C T	[1950]
LMG10241	. . T . . . . . T . . . . . G . . . . T . . . . C . . . . . T . . . . A . . . . G . . .	[1950]
LMG10229	. . T . . . . . T . . . . . G . . A . . . . T . . . . T . . . . A . . . . G . . .	[1950]
LMG9861	. . . . T . . . . . G . . . . T . . . . A	[1950]
LMG9065	. . . . T	[1950]
LMG9871	. . . . T . . . . C	[1950]
LMG 10210	. . . . T . . . . . G . . . . T . . . . A	[1950]
LMG9865	. . . . T . . . . . G . . . . T . . . . A	[1950]
L397	. . T . . . . . T . . . . . G . . A . . . . T . . . . T . . . . C . . . . A	[1950]
L398	. . T . . . . . T . . . . . G . . A . . . . T . . . . C . . . . . C . . . . A	[1950]
L399	. . . . T . . . . . G . . . . T . . . . T . . . . T . . . . A . . . . G . . .	[1950]
L400	. . T . . . . . T . . . . . G . . A . . . . T . . . . T . . . . C . . . . . C . . . . A . . . . G . . .	[1950]
L401	. . . . T . . . . . G . . A T . A . . . . . T . . . . C . . . . A . . . . G . . .	[1950]
L406	. . T . . . . . T . . . . . G . . . . T . . . . C . . . . . T . . . . A . . . . G . . .	[1950]
14PHA	. . . . T . . . . . G . . A . . . . T . . . . T . . . . C . . . . . T . . . . A . . . . G . . .	[1950]
20PHF	. . T . . . . . T . . . . . G . . . . T . . . . C . . . . . T . . . . A . . . . G . . .	[1950]
284/1	. . T . . . . . T . . . . T . . . . C . . . . . C . . . . A	[1950]
8122333	. . T . . . . . T . . . . . G . . . . T . . . . C . . . . . C . . . . A . . . . G . . .	[1950]
8749401	. . T . . . . . T . . . . . G . . A . . . . T . . . . T . . . . C . . . . . C . . . . A . . . . G . . .	[1950]
8756347	. . T . . . . . T . . . . T . . . . T . . . . C . . . . A . . . . G . . .	[1950]
AB3A	. . . . T . . . . . G . . A T . A . . . . . T . . . . C . . . . . C . . . . A . . . . G . . .	[1950]
AB74A	. . T . . . . . T . . . . . G . . A . . . . T . . . . C . . . . . T . . . . A . . . . G . . .	[1950]
AL 20-1	. . . . T	[1950]
AO2A	. . T . . . . . T . . . . . G . . . . T . . . . T . . . . C . . . . . C . . . . A . . . . G . . .	[1950]
BUF3	. . T . . . . . T . . . . . G . . A . . . . T . . . . C . . . . . T . . . . A . . . . G . . .	[1950]
CV-152	. . . . T . . . . T	[1950]
CV-2101	. . . .	[1950]
EMU-3	. . . .	[1950]
F196	. . . . G . . . . T . . . . C . . . . . C . . . . A . . . . G . . .	[1950]
FE 7	. . . . T	[1950]
FEBU4	. . . . G . . A . . . . T . . . . C . . . . . T . . . . A . . . . G . . .	[1950]
HHS 118A	. . T . . . . . T . . . . . G . . . . T . . . . T . . . . C . . . . . C . . . . A . . . . G . . .	[1950]
HHS 133	. . T . . . . . T . . . . . G . . A . . . . T . . . . T . . . . C . . . . . C . . . . A . . . . . C	[1950]
HHS 188A	. . T . . . . . T . . . . . G . . . . T . . . . C . . . . . C . . . . A	[1950]
HHS 191A	. . T . . . . . T . . . . . G . . . . T . . . . T . . . . T . . . . A . . . . G . . .	[1950]
HHS 205A	. . . . G . . A . . . . T . . . . C . . . . . T . . . . A	[1950]
LMG 10829	. . T . . . . . T . . . . A . . . . T . . . . C . . . . A . . . . G . . .	[1950]
LMG 9863	. . . . T	[1950]
MC 2-2	. . . . G . . . . T . . . . C . . . . . T . . . . A . . . . G . . .	[1950]
ME 15-4	. . . . G . . A . . . . T . . . . T . . . . A	[1950]
MICV 42-1	. . T . . . . . T . . . . . G . . A T . A . . . . . T . . . . T . . . . C . . . . A . . . . G . . .	[1950]
NAV 12-2	. . . . G . . . . T . . . . C . . . . A . . . . G . . .	[1950]
NAV 15-1	. . . . T . . . . . G . . . . T . . . . A	[1950]
NB14A	. . T . . . . . T . . . . . G . . . . T . . . . T . . . . C . . . . . C . . . . A . . . . G . . .	[1950]
RW15-1	. . . . T . . . . . G . . . . T . . . . A	[1950]
RW17-4	. . . . T . . . . . G . . . . T . . . . T . . . . A	[1950]
RW25-5	. . . . T . . . . . G . . . . T . . . . A	[1950]
RW33-8	. . T . . . . . T . . . . . G . . . . T . . . . T . . . . C . . . . . C . . . . A . . . . G . . .	[1950]
RW45-3	. . . . T . . . . . G . . . . T	[1950]
UF1T	. . T . . . . . T . . . . . G . . . . T . . . . T . . . . C . . . . . C . . . . A . . . . G . . .	[1950]
UF2T	. . T . . . . . T . . . . . G . . . . T . . . . T . . . . C . . . . . C . . . . A . . . . G . . .	[1950]
UPER3	. . T . . . . . T . . . . . G . . . . T . . . . T . . . . C . . . . . C . . . . A . . . . G . . .	[1950]
A. trophiarum LMG25534T	. . . . . C . . T . . . . . G . . . . T . . . . T . . T . . . . A . . . . . C	[1950]

LMG24291T	T G T G A A G C T A T T T T A G C T G A A C T T A A A A A A G C T T C A A A A G T T G T T G C A A A T A A A A C T G A A A T T G A G C A A G T T G C T A C A	[2028]
LMG10241	. . . . G G . . G . . . . T . . . . A . . . . . A . . . . A . . .	[2028]
LMG10229	. . . . A . . G . . . . T . . . . A . . . . A . . . . . A . . . . G . . .	[2028]
LMG9861	. . . . A . . . . G T . . . . A . . . . G	[2028]
LMG9065	. . . . G . . . . A . . . . A . . . . . A	[2028]
LMG9871	. . . . G . . . . A . . . . A . . . . . A	[2028]
LMG 10210	. . . . A . . . . T . . . . A . . . . G	[2028]
LMG9865	. . . . A . . . . T . . . . A . . . . G	[2028]
L397	. . . . A . . G . . . . T . . . . A . . . . A . . . . . A . . A . . . . A . . .	[2028]
L398	. . . . . G . . . . G G . . G . . . . T . . . . A . . . . G . . . . A . . .	[2028]
L399	. . . . G A . . G . . . . T . . . . A . . . . A . . . . . A . . . . A . . .	[2028]
L400	. . . . A . . G . . . . T . . . . A . . . . . A . . . . A . . .	[2028]
L401	. . . . G G . . G . . . . T . . . . A . . . . . A . . A . . . . A . . .	[2028]
L406	. . . . G G . . G . . . . T . . . . A . . . . . A . . . . . G . . . . . A . . T	[2028]
14PHA	. . . . T A . . G . . . . T . . . . A . . . . . A . . . . . G . . . . . A . . .	[2028]
20PHF	. . . . A . . G . . . . T . . . . A . . . . . A . . . . A . . .	[2028]
284/1	. . . . A . . G . . . . T . . . . A . . . . . A . . . . A . . T	[2028]
8122333	. . . . T A . . G . . . . T . . . . A . . . . . A . . . . . G . . . . . A . . .	[2028]
8749401	. . . . G G . . G . . . . T . . . . A . . . . . A . . . . A . . .	[2028]
8756347	. . . . G A . . G . . . . T . . . . A . . . . . A . . . . A . . .	[2028]
AB3A	. . . . T . . . G . . . . A . . . . A . . . . . A . . . . A . . .	[2028]
AB74A	. . . . G G . . G . . . . T . . . . A . . . . . A . . . . A . . .	[2028]
AL 20-1	. . . . G . . . . A . . . . A . . . . . A	[2028]
AO2A	. . . . G A . . G . . . . T . . . . A . . . . . A . . . . A . . .	[2028]
BUF3	. . . . G G . . G . . . . T . . . . A . . . . . A . . . . A . . .	[2028]
CV-152	. . . .	[2028]
CV-2101	. . . .	[2028]
EMU-3	. . . .	[2028]
F196	. . . . G A . . G . . . . T . . . . A . . . . A . . . . . A . . . . A . . .	[2028]
FE 7	. . . . G . . . . A . . . . A . . . . . A	[2028]
FEBU4	. . . . A . . G . . . . T . . . . . . G . . . . A . . . . . A	[2028]
HHS 118A	. . . . T A . . G . . . . T . . . . A . . . . . A . . . . A . . G	[2028]
HHS 133	. . . . A . . G . . . . T . . . . A . . . . . A . . . . A . . .	[2028]
HHS 188A	. . . . A . . G . . . . T . . . . A . . . . . A . . . . A . . .	[2028]
HHS 191A	. . . . A . . G . . . . T . . . . A . . . . . A	[2028]
HHS 205A	. . . . A . . G . . . . T . . . . A . . . . . A . . . . A . . .	[2028]
LMG 10829	. . . . A . . G . . . . T . . . . A . . . . . A . . . . . G . . . . . A . . .	[2028]
LMG 9863	. . . . G . . . . A . . . . A . . . . . A	[2028]
MC 2-2	. . . . T A . . G . . . . T . . . . A . . . . . A . . . . A . . G	[2028]
ME 15-4	. . . . G . . . . A . . . . A . . . . . A	[2028]
MICV 42-1	. . . . T . A . . G . . . . T . . . . G . . . . A . . . . . A . . A . . . . A . . .	[2028]
NAV 12-2	. . . . A . . G . . . . T . . . . A . . . . . A . . A	[2028]
NAV 15-1	. . . . A . . . . T . . . . A . . . . G	[2028]
NB14A	. . . . G A . . G . . . . T . . . . A . . . . . A . . . . A . . .	[2028]
RW15-1	. . . . A . . . . T . . . . A . . . . G	[2028]
RW17-4	. . . . A . . . . T . . . . A . . . . G	[2028]
RW25-5	. . . . A . . . . T . . . . A . . . . G	[2028]
RW33-8	. . . . G A . . G . . . . T . . . . A . . . . . A . . . . A . . .	[2028]
RW45-3	. . . . C . . . . A . . . . T . . . . A . . . . G	[2028]
UF1T	. . . . G A . . G . . . . T . . . . A . . . . . A . . . . A . . .	[2028]
UF2T	. . . . G G . . G . . . . T . . . . A . . . . . A . . . . . G . . . . . A . . .	[2028]
UPER3	. . . . A . . G . . . . T . . . . A . . . . A . . . . . A . . . . A . . .	[2028]
A. trophiarum LMG25534T	. . . . A . . . . T . . . . G . G . . . . A	[2028]

LMG24291T	A T T T C T G C A A A C T C T G A T A G T G C A A T T G G A A A A A T G A T T G C T G A A G C T A T G G A A A A A G T T G G A A A A G A T G G A G T T A T A	[2106]
LMG10241	. . . . . A . . . . A . . . . T . . . . . T	[2106]
LMG10229	. . . . A . . . . T . . . . T . . . . . T	[2106]
LMG9861	. . . .	[2106]
LMG9065	. . . . . A . . . . C . . T	[2106]
LMG9871	. . . . . A . . . . A . . . . T . . . . . T	[2106]
LMG 10210	. . . .	[2106]
LMG9865	. . . .	[2106]
L397	. . . . . A . . . . A . . . . T . . . . . T	[2106]
L398	. . . . . A . . . . A . . . . T . . . . . T	[2106]
L399	. . . . . A . . . . A . . . . T . . . . . T	[2106]
L400	. . . . . A . . . . A . . . . T . . . . . T	[2106]
L401	. . . . . A . . . . A . . . . T . . . . . T	[2106]
L406	. . C . . . . A . . . . C . . . . T . . . . . T	[2106]
14PHA	. . . . . A . . . . A . . . . T . . . . . T	[2106]
20PHF	. . . . . A . . . . A . . . . . C . . . . T . . . . . T	[2106]
284/1	. . C . . . . A . . . . T . . . . . T	[2106]
8122333	. . . . . A . . . . A . . . . T . . . . . T	[2106]
8749401	. . . . . A . . . . A . . . . . C . . . . T . . . . . T	[2106]
8756347	. . . . . A . . . . A . . . . T . . . . . T	[2106]
AB3A	. . . . . A . . . . A . . . . T	[2106]
AB74A	. . . . . A . . . . A . . . . T . . . . . T	[2106]
AL 20-1	. . . . . A . . . . A . . . . C . . T . . . . . T	[2106]
AO2A	. . . . . A . . . . A . . . . T . . . . . T	[2106]
BUF3	. . . . . A . . . . A . . . . . C . . . . T . . . . . T	[2106]
CV-152	. . . .	[2106]
CV-2101	. . . .	[2106]
EMU-3	. . . .	[2106]
F196	. . . . . A . . . . A . . . . T . . . . . T	[2106]
FE 7	. . . . . A . . . . A . . . . C . . T . . . . . T	[2106]
FEBU4	. . . . . A . . . . A . . . . T . . . . . T	[2106]
HHS 118A	. . . . . A . . . . A . . . . T . . . . . T	[2106]
HHS 133	. . . . . A . . . . A . . . . T	[2106]
HHS 188A	. . . . . A . . . . A . . . . T . . . . . T	[2106]
HHS 191A	. . . . . A . . . . A . . . . T . . . . . T	[2106]
HHS 205A	. . . . . A . . . . A . . . . T . . . . . T	[2106]
LMG 10829	. . . . . A . . . . A . . . . A . . . . T . . . . . T	[2106]
LMG 9863	. . . . . A . . . . C . . T . . . . . T	[2106]
MC 2-2	. . . . . A . . . . A . . . . T . . . . . T	[2106]
ME 15-4	. . . . . A . . . . A . . . . C . . T . . . . . T	[2106]
MICV 42-1	. . . . . A . . . . A . . . . T . . . . T . . . . . T	[2106]
NAV 12-2	. . . . . A . . . . A . . . . T . . . . . T	[2106]
NAV 15-1	. . . .	[2106]
NB14A	. . . . . A . . . . A . . . . T . . . . . T	[2106]
RW15-1	. . . .	[2106]
RW17-4	. . . .	[2106]
RW25-5	. . . .	[2106]
RW33-8	. . . . . A . . . . A . . . . T . . . . . T	[2106]
RW45-3	. . . .	[2106]
UF1T	. . . . . A . . . . A . . . . . C . . . . T . . . . . T	[2106]
UF2T	. . . . . A . . . . A . . . . T . . . . . T	[2106]
UPER3	. . . . . A . . . . A . . . . T . . . . . T	[2106]
A. trophiarum LMG25534T	. . . . G . . . . G . . . . T	[2106]

LMG24291T	A C T G T T G A A G A G G C A A A A G G T A T T T C A G A T G A A C T T G A T G T T G T A G A G G G A A T G C A G T T T G A T A G A G G A T A T T T A A G T	[2184]
LMG10241	. . . . A . . . . . G . . . . A . . . . T . . A	[2184]
LMG10229	. . . . A . . . . A . . . . G	[2184]
LMG9861	. . . .	[2184]
LMG9065	. . . . A . . . . G . . . . T . . A	[2184]
LMG9871	. . . . A . . . . G . . . . T . . A	[2184]
LMG 10210	. . . . A	[2184]
LMG9865	. . . .	[2184]
L397	. . . . T . . . . . A . . . . A . . . . A	[2184]
L398	. . . . A . . . . . G . . . . . G . . A . . . . T . . A	[2184]
L399	. . . . A . . . . . G . . . . A . . . . A	[2184]
L400	. . . . A . . . . . T . . . . . G . . A . . . . A	[2184]
L401	. . . . A . . . . . G . . . . A . . . . A	[2184]
L406	. . . . A . . . . . G . . . . A . . . . A	[2184]
14PHA	. . . . A . . . . . T . . . . . G . . A . . . . A	[2184]
20PHF	. . . . A . . . . . T . . . . . G . . A . . . . A	[2184]
284/1	. . . . A . . C . . T . . C . . G . . A . . . . A	[2184]
8122333	. . . . A . . . . . G . . . . . G . . A . . . . A . . . . A	[2184]
8749401	. . . . A . . . . . G . . . . A . . . . A	[2184]
8756347	. . . . A . . . . . T . . . . A . . . . A . . . . A	[2184]
AB3A	. . . . A . . . . . G . . . . A	[2184]
AB74A	. . . . A . . . . . G . . . . A . . . . A	[2184]
AL 20-1	. . . . A . . . . G . . . . T . . A	[2184]
AO2A	. . . . A . . . . . G . . . . . G . . A . . . . A . . . . A	[2184]
BUF3	. . . . A . . . . . G . . . . A . . . . A	[2184]
CV-152	. . . .	[2184]
CV-2101	. . . .	[2184]
EMU-3	. . . .	[2184]
F196	. . . . T . . . . C . . T . . . . . G T . A	[2184]
FE 7	. . . . A . . . . C . . . . G . . . . T . . A	[2184]
FEBU4	. . . . A . . . . . G . . . . . G . . A	[2184]
HHS 118A	. . . . A . . . . . G . . . . A . . . . A	[2184]
HHS 133	. . . . A . . . . A . . . . A	[2184]
HHS 188A	. . . . A . . . . . G . . . . . G . . A . . . . T . . A	[2184]
HHS 191A	. . . . A . . . . . T . . . . . G . . A . . . . A	[2184]
HHS 205A	. . . . A . . . . . G . . . . A	[2184]
LMG 10829	. . . . A . . C . . T . . C . . . . . A . . . . A	[2184]
LMG 9863	. . . . A . . . . G . . . . T . . A	[2184]
MC 2-2	. . . . A . . . . . G . . . . A . . C . . . . A	[2184]
ME 15-4	. . . . A . . . . C . . . . G . . . . T . . A	[2184]
MICV 42-1	. . . . A . . . . A . . . . . T . . . . A . . . . A	[2184]
NAV 12-2	. . . . A . . . . . G . . . . A	[2184]
NAV 15-1	. . . .	[2184]
NB14A	. . . . A . . . . . G . . . . . G . . A . . . . A . . . . A	[2184]
RW15-1	. . . .	[2184]
RW17-4	. . . .	[2184]
RW25-5	. . . .	[2184]
RW33-8	. . . . A . . . . . G . . . . . G . . A . . . . A . . . . A	[2184]
RW45-3	. . . . A	[2184]
UF1T	. . . . A . . . . . G . . . . A . . C . . . . A	[2184]
UF2T	. . . . A . . . . . G . . . . A . . C . . . . A	[2184]
UPER3	. . . . A . . . . . G . . . . A . . . . A	[2184]
A. trophiarum LMG25534T	. . A . . . . A . . . . A . . . . . T . . . . A . . . . T . . A	[2184]

```
LMG24291T                   CCATATTTTGTAACAAATCCTGAAAAAATGACAACAGAGTTTAATAATCCATTTATTTTACTTTATGATAAAAAAATC [2262]
LMG10241                    ...........T................................C............C...................A [2262]
LMG10229                    ...........T................................C............C...................A [2262]
LMG9861                     ...........T.................................................................. [2262]
LMG9065                     ...........T................................C................................. [2262]
LMG9871                     ...........T................................C................................A [2262]
LMG 10210                   ...........T.................................................................. [2262]
LMG9865                     .............................................................................. [2262]
L397                        ...........T..........................T..................C...................A [2262]
L398                        ...........T................................C............C...................A [2262]
L399                        ...........T................................C............C...................A [2262]
L400                        ...........T................................C............C...................A [2262]
L401                        ...........T................................C............C...................A [2262]
L406                        ...........T.............................................C....A............... [2262]
14PHA                       ...........T................................C............C...................A [2262]
20PHF                       ...........T.............................................C...................A [2262]
284/1                       ...........T.............................................C....A............... [2262]
8122333                     ...........T................................C............C...................A [2262]
8749401                     ...........T................................C............C...................A [2262]
8756347                     ...........T................................C............C...................A [2262]
AB3A                        ...........T................................C............C...................A [2262]
AB74A                       ...........T.............................................C...................A [2262]
AL 20-1                     ...........T................................C................................. [2262]
AO2A                        ...........T.............................................C...................A [2262]
BUF3                        ...........T.............................................C.......C............ [2262]
CV-152                      .............................................................................. [2262]
CV-2101                     .............................................................................. [2262]
EMU-3                       .............................................................................. [2262]
F196                        ...........T................................C............C...................A [2262]
FE 7                        ...........T................................C................................. [2262]
FEBU4                       ...........T.............................................C...................A [2262]
HHS 118A                    ...........T................................C............C...................A [2262]
HHS 133                     ...........T................................C............C...................A [2262]
HHS 188A                    ...........T................................C............C...................A [2262]
HHS 191A                    ...........T................................C............C...................A [2262]
HHS 205A                    ...........T.............................................C...................A [2262]
LMG 10829                   ...........T................................C............C...................A [2262]
LMG 9863                    .............................................................................A [2262]
MC 2-2                      ...........T................................C............C...................A [2262]
ME 15-4                     ...........T................................C................................. [2262]
MICV 42-1                   ...........T.............................................C...................A [2262]
NAV 12-2                    ...........T.............................................C.G.................A [2262]
NAV 15-1                    ...........T.................................................................. [2262]
NB14A                       ...........T.............................................C...................A [2262]
RW15-1                      .............................................................................. [2262]
RW17-4                      ...........T.................................................................. [2262]
RW25-5                      .............................................................................. [2262]
RW33-8                      ...........T.............................................C...................A [2262]
RW45-3                      .............................................................................. [2262]
UF1T                        ...........T................................C............C...................A [2262]
UF2T                        ...........T................................C............C...................A [2262]
UPER3                       ...........T................................C............C...................A [2262]
A. trophiarum LMG25534T     ....................A.....................G.......T.....CC.TT.A............... [2262]
```

LMG24291T	T C T T C A T T A A A A G A G A T G T T A C C A A T T T T A G A A G G T G T T A A T A A A T C T G G A A G A C C T C T T T T A A T C A T T G C T G A A G A T	[2340]
LMG10241	. . . . T . . . . A . . . . . A	[2340]
LMG10229	. . . . T . . . . A . . . . . A	[2340]
LMG9861	. . . . A . . . . A	[2340]
LMG9065	. . . . A C . T . . . . T . A . . . . . T	[2340]
LMG9871	. . . . A C . T . . . . G . . . . T . A . . . . . T	[2340]
LMG 10210	. . . . A . . . . A	[2340]
LMG9865	. . . . A	[2340]
L397	. . . . G . . T . . . . A . . . . . A	[2340]
L398	. . . . G . . T . . . . A	[2340]
L399	. . . . G . . T . . . . T	[2340]
L400	. . . . . . C . . . . C . T . . . . A C . T . . . . G . . T	[2340]
L401	. . . . . G . . . . T . . . . A . . . . . A	[2340]
L406	. . . . . . C . . . . C . T . . . . . T . . . . A . . . . . A	[2340]
14PHA	. . . . T . . . . A . . . . . A	[2340]
20PHF	. . . . T . . . . T . . . . . A	[2340]
284/1	. . . . . . C . . . . C . T . . . . A C . . . . A . . . . . A	[2340]
8122333	. . . . T . . . . A . . . . . A	[2340]
8749401	. . . . T . . . . T	[2340]
8756347	. . . . T . . . . T . . . . . A	[2340]
AB3A	. . . . T . . . . A . . . . . A	[2340]
AB74A	. . . . T . . . . A . . . . . A	[2340]
AL 20-1	. . . . A C . T . . . . T . A . . . . . T	[2340]
AO2A	. . . . T . . . . T . . . . . A	[2340]
BUF3	. . . . . . C . . . . C . T . . . . A C . . . . A . . . . . . G . A . . . C . T . . A . . . . T	[2340]
CV-152	. . . . A	[2340]
CV-2101	. . . .	[2340]
EMU-3	. . . .	[2340]
F196	. . . . T . . . . A . . . . . A	[2340]
FE 7	. . . . A C . T . . . . T . A . . . . . T	[2340]
FEBU4	. . . . T . . . . A . . . . . A	[2340]
HHS 118A	. . . . . . C . . . . C . T . . . . A C . T . . . . T	[2340]
HHS 133	. . . . T . . . . A . . . . . A	[2340]
HHS 188A	. . . . G . . T . . . . A . . C . . A	[2340]
HHS 191A	. . . . T . . . . T	[2340]
HHS 205A	. . . . G . . T . . . . A . . . . . A	[2340]
LMG 10829	. . . . T . . . . T . . . . . A	[2340]
LMG 9863	. . . . A C . T . . . . T . A . . . . . T	[2340]
MC 2-2	. . . . T . . . . T	[2340]
ME 15-4	. . . . A C . T . . . . T . A . . G . . T	[2340]
MICV 42-1	. . . . C . T . . . . A C . T . . . . A . . . . . A	[2340]
NAV 12-2	. . . . G . . T . . . . T	[2340]
NAV 15-1	. . . . A	[2340]
NB14A	. . . . T . . . . T . . . . . A	[2340]
RW15-1	. . . . A	[2340]
RW17-4	. . . . A	[2340]
RW25-5	. . . . A	[2340]
RW33-8	. . . . T . . . . T . . . . . A	[2340]
RW45-3	. . . . A	[2340]
UF1T	. . . . . . C . . . . T . . . . A . . . . . A	[2340]
UF2T	. . . . T . . . . T . . . . . A	[2340]
UPER3	. . . . T . . . . T . . . . . A	[2340]
A. trophiarum LMG25534T	. . A . . T . . . . C . A . . . . C . T . . . . A . . . . . . G . . . . . C . T . . A . . . . T G . A	[2340]

LMG24291T	G T T G A T G G T G A A G C A C T A G C A A C A C T T G T T G T A A A T A G A T T A A G A G G C G C A T T A C A A A T T G C A G C T G T	[2408]
LMG10241	. . . . T . . . . T . . T T . G . . . . T	[2408]
LMG10229	. . A . . . . T . . . . G . . T T . G . . . . A . . . . T	[2408]
LMG9861	. . . . T T . . . . T T . G . . . . T . . . . T . . A . .	[2408]
LMG9065	. . A . . . . T . . . . T	[2408]
LMG9871	. . A . . . . T . . . . . T T . G . . . . C . . . . T	[2408]
LMG 10210	. . . . T T . . . . T . . T T . G . . . . T . . . . T . . A . .	[2408]
LMG9865	. . . . T T . . . . T T . G . . . . T . . . . T . . A . .	[2408]
L397	. . . . T . . . . T T . A . . . . T . . . . T	[2408]
L398	. . . . T . . . . T T . G . . . . T	[2408]
L399	. . . . T . . . . T T . A . . . . T . . . . T	[2408]
L400	. . . . T . . . . T T . G . . . . T	[2408]
L401	. . . . T . . . . T T . A . . . . T . . . . T	[2408]
L406	. . . . T . . . . T T . A . . . . T . . . . T	[2408]
14PHA	. . . . T . . . . T T . A . . . . T . . . . T	[2408]
20PHF	. . . . T . . . . T T . A . . . . T	[2408]
284/1	. . . . T . . . . T T . A . . . . T . . . . T	[2408]
8122333	. . . . T . . . . T . . T T . G . . . . T	[2408]
8749401	. . . . T . . . . T . . T T . G . . . . T	[2408]
8756347	. . . . T . . . . T T . G . . . . T . . . . T	[2408]
AB3A	. . . . T . . . . T T . A . . . . T . . . . T	[2408]
AB74A	. . . . T . . . . T T . A . . . . T . . . . T	[2408]
AL 20-1	. . A . . . . T . . . . T	[2408]
AO2A	. . . . T . . . . T T . A . . . . T	[2408]
BUF3	. . . . T . . . . T T . A . . . . T . . . . T	[2408]
CV-152	. . . .	[2408]
CV-2101	. . . . T	[2408]
EMU-3	. . . . T	[2408]
F196	. . . . T . . . . T T . A . . . . T . . . . T	[2408]
FE 7	. . A . . . . T . . . . T	[2408]
FEBU4	. . A . . . . T . . . . G . . T T . G . . . . T	[2408]
HHS 118A	. . . . T . . . . T T . A . . . . T . . . . T	[2408]
HHS 133	. . A . . . . G . . . T . . . . G . . T T . G . . . . T	[2408]
HHS 188A	. . . . T . . . . T . . T T . G . . . . T	[2408]
HHS 191A	. . . . T . . . . T T . A . . . . T . . . . T	[2408]
HHS 205A	. . A . . . . T . . . . G . . T T . G . . . . T	[2408]
LMG 10829	. . . . T . . . . T T . G . . . . T . . . . T	[2408]
LMG 9863	. . A . . . . T . . . . T	[2408]
MC 2-2	. . . . T . . . . T . . T T . G . . . . T . . . . T	[2408]
ME 15-4	. . A . . . . T . . . . T	[2408]
MICV 42-1	. . . . T . . . . T T . A . . . . T . . . . T	[2408]
NAV 12-2	. . A . . . . T . . . . T T . A . . . . T . . . . T	[2408]
NAV 15-1	. . . . T T . . . . T T . G . . . . T . . . . T . . A . .	[2408]
NB14A	. . . . T . . . . T T . A . . . . T	[2408]
RW15-1	. . . . T T . . . . T T . G . . . . T . . . . T . . A . .	[2408]
RW17-4	. . . . T T . . . . T . . T T . G . . . . T . . . . T . . A . .	[2408]
RW25-5	. . . . T T . . . . T T . G . . . . T . . . . T . . A . .	[2408]
RW33-8	. . . . T . . . . T T . A . . . . T	[2408]
RW45-3	. . . . T T . . . . T T . G . . . . T . . . . T . . A . .	[2408]
UF1T	. . . . T . . . . T T . G . . . . T . . . . T	[2408]
UF2T	. . . . T . . . . T T . A . . . . T	[2408]
UPER3	. . . . T . . . . T T . A . . . . T . . . . T	[2408]
A. trophiarum LMG25534T	. . A . . . . . A . . . . . T . . T . . . . . T T . A . . . . G . . T . . . . T	[2408]
